# A cub and sushi domain-containing protein with esterase-like activity confers insecticide resistance in the Indian malaria vector *Anopheles stephensi*

**DOI:** 10.1016/j.jbc.2024.107759

**Published:** 2024-09-10

**Authors:** Jatin Kumar, Ankit Kumar, Yash Gupta, Kapil Vashisht, Shivam Kumar, Arvind Sharma, Raj Kumar, Ashoke Sharon, Praveen K. Tripathi, Ram Das, Om Prakash Singh, Shailja Singh, Soumyananda Chakraborti, Sujatha Sunil, Kailash C. Pandey

**Affiliations:** 1 ICMR- National Institute of Malaria Research, New Delhi, India; 2 Academy of Scientific and Innovative Research (AcSIR), Ghaziabad, Uttar Pradesh, India; 3 International Centre for Genetic Engineering and Biotechnology, New Delhi, India; 4 Department of Medicine, Penn State College of Medicine, Hershey, Pennsylvania, USA; 5 Department of Chemistry, Birla Institute of Technology-Mesra, Ranchi, Jharkhand, India; 6 Special Centre for Molecular Medicine, Jawaharlal Nehru University, New Delhi, India

**Keywords:** malaria, *Anopheles stephensi*, insecticide resistance, esterase-like protein, silencing

## Abstract

Chemical insecticides (organophosphates and pyrethroids) in the form of IRS (Indoor Residual Sprays) and LLINs (Long Lasting Insecticidal Nets) are the cornerstone for vector control, globally. However, their incessant use has resulted in widespread development of resistance in mosquito vectors, warranting continuous monitoring and investigation of the underlying mechanisms of resistance. Here, we identified a previously uncharacterized- Cub and Sushi Domain containing Insecticide Resistance (CSDIR) protein and generated evidence for its role in mediating insecticide resistance in the *Anopheles stephensi*. A strong binding affinity of the CSDIR protein towards different classes of insecticide molecules-malathion (K_D_ 6.43 μM) and deltamethrin (K_D_ 46.7 μM) were demonstrated using MD simulation studies and Surface Plasmon Resonance (SPR) experiments. Further, the recombinant CSDIR^913-1190^ protein exhibited potent esterase-like activity (α-naphthyl acetate (α-NA)- 1.356 ± 0.262 mM/min/mg and β-naphthyl acetate (β -NA)- 1.777 ± 0.220 mM/min/mg). Interestingly, dsRNA-mediated gene silencing of the CSDIR transcripts caused >60% mortality in resistant *An. stephensi* upon 1-h exposure to deltamethrin and malathion insecticides, compared to the control group. A significant reduction in the esterase-like activity was also observed against α-NA (*p* = 0.004) and β-NA (*p* = 0.025) in CSDIR silenced mosquitoes compared to the control group. Using computational analysis and experimental data, our results provided significant evidence of the involvement of the CSDIR protein in mediating insecticide resistance in *Anopheles* mosquitoes. Thereby making the CSDIR protein, a novel candidate for exploration of novel insecticide molecules. These data would also be helpful in further understanding the development of metabolic resistance by the *Anopheles* vector.

According to World Malaria Report 2022, an estimated 247 million malaria cases were reported globally. Among these, India accounted for 79% of the cases in the WHO South-East Asia region (1). Malaria vector control in India is exclusively based on chemical insecticides used in Long-Lasting Insecticidal Nets (LLINs) and Indoor Residual Sprays (IRS) (2). Pyrethroids and organophosphates are the most widely used classes of insecticides in LLINs and IRS, respectively. However, their widespread and incessant use in vector control has led to prevalent resistance in all mosquito species (3, 4). In addition, the non-target effects of these insecticides on the environment cannot be neglected (5, 6). According to the World Malaria Report 2021, 87% and 60% of the countries have reported resistance against pyrethroids and organophosphates, respectively (2). In the Indian context, confirmed resistance against organophosphates and pyrethroids has been reported for major malaria vectors- *An. culicifacies* and *An. stephensi* (7). Major mechanisms of insecticide resistance include target-site insensitivity (knockdown resistance, *kdr*) and metabolic resistance through over-expression of detoxification enzymes (cytochrome P450s- CYPs, glutathione S-transferases- GSTs, and esterases) (8, 9). Several cytochrome P450 genes like *CYP9K1*, *CYP6P3*, *CYP6M2*, *CYP6Z1* (10), and *CYP325A3*, were reportedly found to be associated with pyrethroid resistance in the *Anopheles* mosquitoes (11). Recently, an association of the *CYP6P9a* gene with pyrethroid resistance was seen, imposing a fitness cost in the African malaria vector *An. funestus* (12). Transcriptome study on pyrethroid-resistant *An. gambiae* found overexpression of genes of well-known detoxifying enzyme family such as *CYP6Z3* (AGAP008217), *GSTD1* (AGAP004164), *GSTD7* (AGAP004163), *GSTD3* (AGAP004382), *GSTE5* (AGAP009192), *GSTMS3* (AGAP009946), *COEAE8O* (AGAP006700), *CYP4C28* (AGAP010414), *CYP12F2* (AGAP008020). Additionally, novel candidate genes such as α-crystallins and hexamerins were also found associated with insecticide resistance. Silencing these genes imparted significant mortality after deltamethrin exposure in the pyrethroid-resistant *An. gambiae* (13). Similarly, the upregulation of genes belonging to the ABC transporters was also found to mediate pyrethroid resistance in *An. gambiae* (14). A chemosensory protein-sensory appendage protein 2 (SAP2), enriched in the mosquito legs has been shown to confer pyrethroid resistance in *An. gambiae* through insecticide sequestration (15).

While multiple studies from around the globe are available on understanding insecticide resistance in mosquitoes (3), studies on Indian malaria vector- *An. stephensi* are lacking, particularly metabolic resistance. We set out to explore the status of insecticide resistance in *An. stephensi* mosquitoes and found a previously uncharacterized protein (XP_035915664.1), that mediates insecticide resistance through esterase-like activities. Using computational and biochemical tools, we demonstrated XP_035915664.1 strongly interacted with two different classes of insecticides-deltamethrin (pyrethroid) and malathion (organophosphate). Further, RNAi-mediated knockdown of the XP_035915664.1 transcripts resulted in enhanced toxicity in the deltamethrin/malathion-resistant mosquitoes.

## Results

### Identification of differentially expressed proteins from deltamethrin and malathion-resistant *A. stephensi*

**Figure 1:**
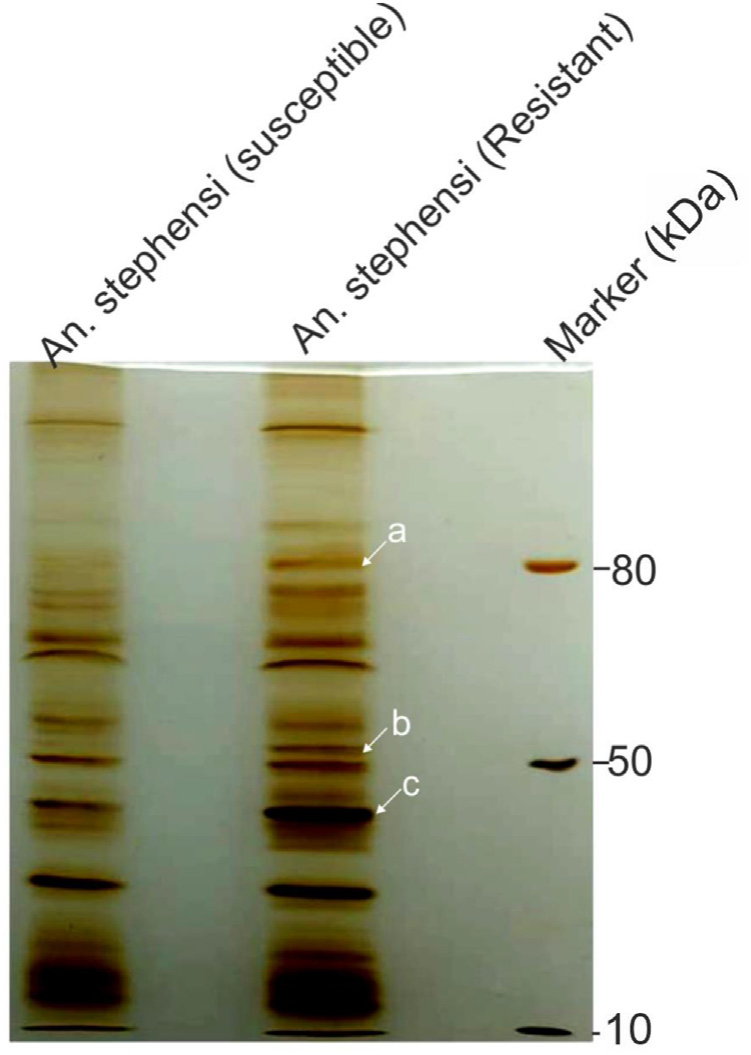
**Identification of CSDIR protein from Resistant *An. stephensi*.** Silver stained SDS-PAGE gel showing susceptible and resistant female *An. stephensi* lysate. Highlighted bands *a*, *b*, *c* from resistant *An. stephensi* lysate were selected for LC/MS-MS analysis.

The standard adult susceptibility assay for deltamethrin and malathion was performed on the *Anopheles stephensi* strain from Chennai and, Mewat regions of India, which were adopted in the insectary facility at ICMR-NIMR, Delhi. Chennai line showed 100% mortality and considered susceptible to both malathion and deltamethrin molecules while Mewat line showed 15 ± 3.31% mortality against malathion and 21 ± 3.31% mortality against deltamethrin and therefore, considered resistant to both molecules (Fig. S1). Three differentially expressing protein bands (*a*, *b*, *c*) were observed from comparison of deltamethrin/malathion (resistant & susceptible) *An. stephensi* mosquito lysates on SDS-PAGE, selected bands were further subjected to LC/MS-MS analysis (Fig. 1) and identified proteins are listed in Table 1. In resistant mosquitoes, band *c* had highest level of differential protein expression. Band *a* contained two protein hits-the H4 Histone protein of *Culex quinquefasciatus* with a role in DNA replication and a hypothetical protein of *An. sinensis* of unknown function (16). Band *b* identified dehydrogenase/reductase SDR family member of *An. stephensi* with putative role as oxidoreductase (17). LC/MS-MS analysis of band *c* found three uncharacterized proteins-, 14-3-3 protein epsilon, and pyruvate dehydrogenase E1 component subunit-β, mitochondrial (*An. stephensi)* (18), and uncharacterized protein (XP_035915664.1). The uncharacterized protein (XP_035915664.1) had highest sequence coverage in the LC/MS-MS data (Table 1), and therefore, chosen for further investigation.

**Table 1:** Identified proteins after LC/MS-MS sequencing from the selected bands

Band code	NCBI IDs	Sequence coverage	Protein name	Reference
*a*	EDS37346.1	47	H4 Histone protein (*Culex quinquefasciatus*)	DNA Replication (16)
	KFB50208.1	43	hypothetical protein ZHAS_00018276 (*Anopheles sinensis*)	N/A
*b*	XP_035899120.1	45	dehydrogenase/reductase SDR family member on chromosome X-like (*Anopheles stephensi*).	NAD(P) (H)- dependent oxidoreductases (17)
*c*	XP_035915664.1	34	uncharacterised protein (*Anopheles stephensi*)	N/A
	XP_035919672.1	31	14-3-3 protein epsilon (*Anopheles stephensi*)	N/A
	XP_035901711.1	31	pyruvate dehydrogenase E1 component subunit beta, mitochondrial (*Anopheles stephensi*)	Glycolysis (18)

### Computational analysis of the XP_035915664.1 protein

**Figure 2:**
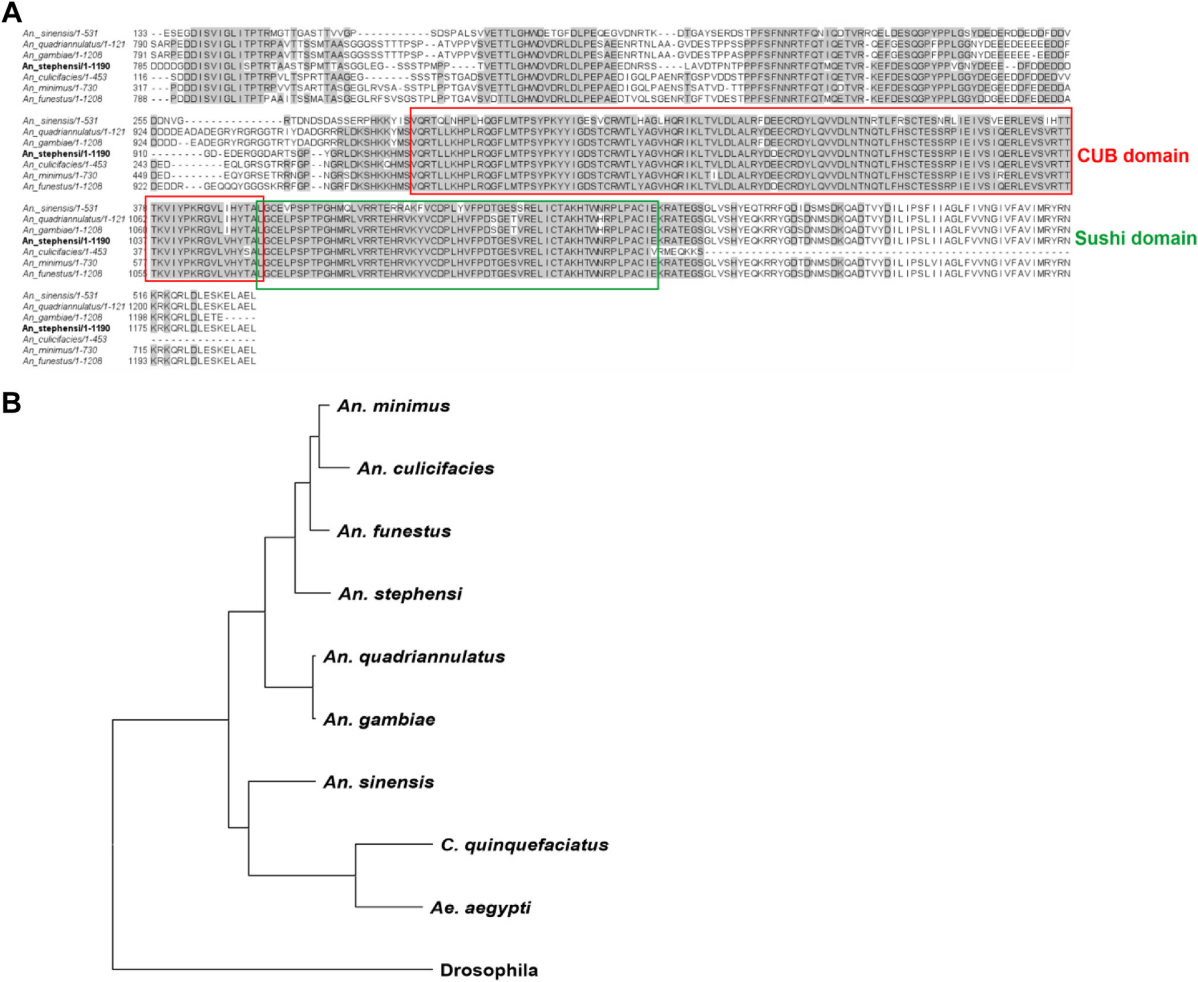
**MultipleSequence Alignment and Phylogenetic tree of CSDIR protein.***A*, partial Multiple Sequence Alignment of CSDIR with different *Anopheles* species showing highly conserved CUB and sushi domains. *B*, phylogenetic tree of CSDIR from different species of *Anopheles.*

**Figure 3:**
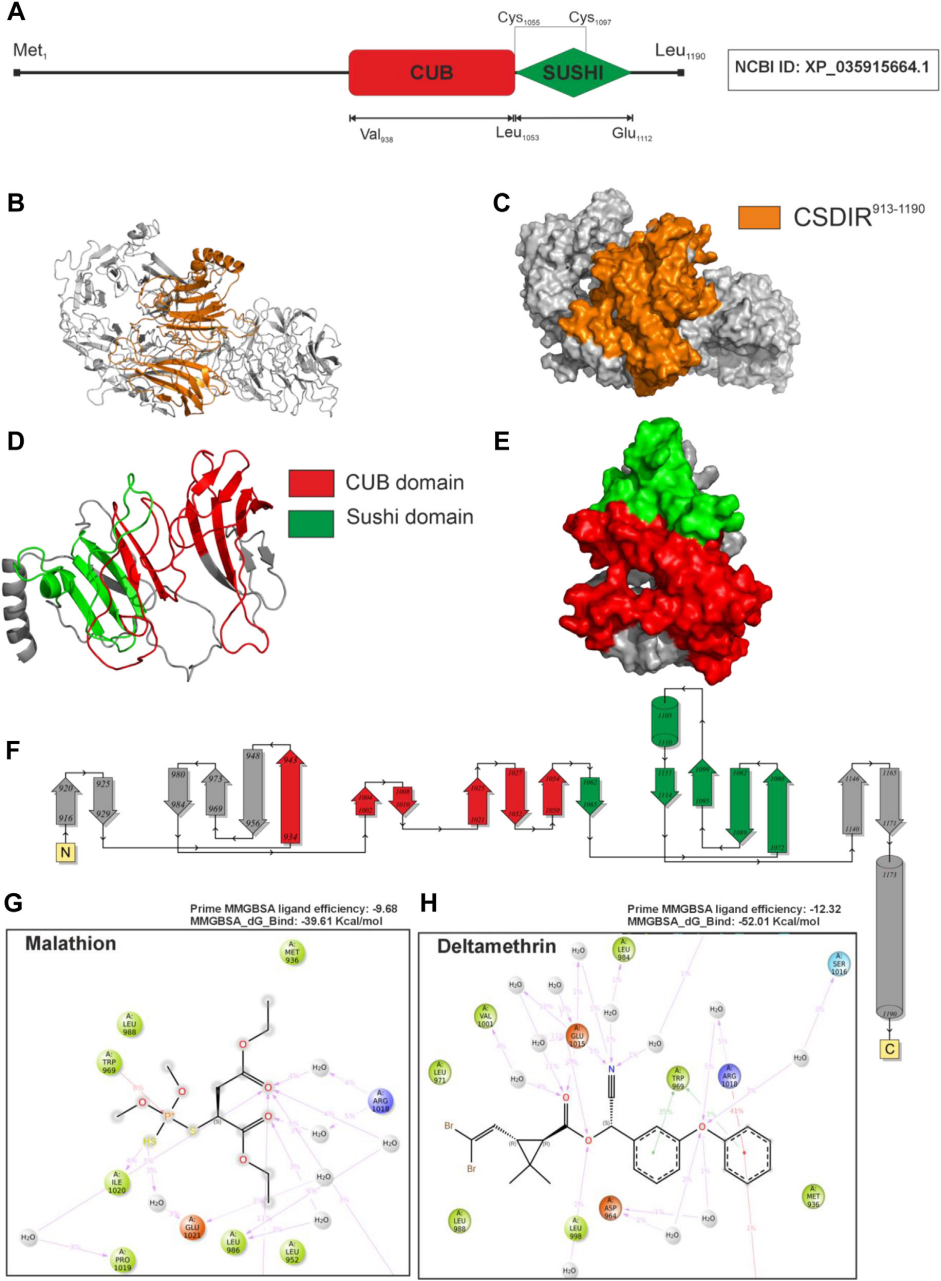
**Computational analysis of the CSDIR protein.***A*, domain annotation of CSDIR protein. *B*, cartoon representing the CSDIR protein molecular structure, the C-terminal cloned region is highlighted in orange color. *C*, surface 3D model of the CSDIR protein, the C-terminal cloned region is highlighted in orange color. *D*, cartoon representing CSDIR^913-1190^ protein molecular structure. *E*, surface 3D model of the CSDIR^913-1190^ protein, the CUB and sushi domains are highlighted in red and green color, respectively. *F*, topology diagram of CSDIR^913-1190^ protein, arrows representing β-sheets, cylinders representing α-helices. *G*, zoomed in protein-ligand interaction map showing schematic 2D molecular interaction of CSDIR amino acid residues bound to malathion. *H,* zoomed in protein-ligand interaction map showing schematic 2D molecular interaction of CSDIR amino acid residues bound to deltamethrin.

The Multiple Sequence Alignment (MSA) of the XP_035915664.1 protein with other major malaria vectors (*An. sinensis, An. quadriannulatus, An. minimus, An. funestus, An. culicifacies and An. stephensi*) found highly conserved CUB and Sushi domains at the C-terminal region (Fig. 2*A*). For the presence of these domains, we shall annotate this protein as CUB and Sushi Domain-containing Insecticide Resistance (CSDIR) protein, further on in this manuscript. Additionally, we created a CSDIR protein phylogenetic tree showing the evolutionary relationship of the protein with the orthologues in *Anopheles*, *Aedes*, *Culex* and *Drosophila* (Fig. 2*B*). The phylogenetic tree analysis indicated *drosophila* as the outlier group, whereas the culicine and anopheline species formed separate clades. Among the anophelines, *An. quadriannulatus* and, *An. gambiae* (African Vectors), were found in a separate clade, whereas Asian Vectors-*An. stephensi*, *An. culicifacies*, *An. minimus* formed a separate clade. Interestingly, *An. funestus* (African Vector) grouped with the Asian vectors; corroborating the findings of *Neafsey et al. 1979* (19). Furthermore, we calculated the Ka/Ks ratio to estimate nonsynonymous/substitution mutation (Ka) and synonymous/silent mutation (Ks) rates in the CSDIR protein sequence. The overall Ka/Ks ratio was found to be 0.20, indicating that CSDIR protein might not undergoing a positive selection. However, we found some amino acid residues that can potentially undergo positive selection in future (Fig. S2). Through domain prediction analysis, we marked CUB and Sushi domains at the C-terminal region of the uncharacterized protein Figure 3*A*. Since, the overexpressing protein band *c* from resistant *An. stephensi* comprised-the CSDIR protein, it is imperative to investigate any potential contribution of the uncharacterized CSDIR protein in mediating insecticide resistance. To further investigate molecular interactions (*in silico*) of the CSDIR protein with insecticides-deltamethrin and malathion, a full-length structural model of this protein was generated through homology modelling using I-TASSER online server (Fig. 3, *B* and *C*) (20, 21, 22). We also compared I-TASSER generated model with the AlphaFold3 (23). Most of the models generated by AlphaFold3 were in low confidence range and remained unmodelled. This observation made the output unsuitable for structural studies. The limited success of CSDIR modeling by AlphaFold3 can be largely due to the lack of coevolution signal, demonstrated by the lack of effect of MSA input, *versus* structural or geometric features of subdomains. There are reports citing the importance of MSAs and coevolution signals in AlphaFold's global conformational search (24). The ipTM score was 0.36 vs. the acceptable threshold of approximately 0.75 (25). For a reliable model for CSDIR generated from AI-based tools, an AI-tool trained with sufficient insect proteins and enzymes of this class from diverse phyla is needed. This also emphasizes an urgent requirement for crystallization and 3D microscopy structural determination of proteins from different phyla. A detailed comparison can be visualised in Fig. S9. However, due to the low confidence score of the AlphaFold3 model, we preferred I-TASSER model for further analysis. Due to the presence of functional domains at C-terminal region, we chose residues (CSDIR^913-1190^) for recombinant protein production and, further investigation. A 3D model of CSDIR^913-1190^ region is shown in Figure 3, *D* and *E*. The topology was calculated using the PDBSUM server (http://www.ebi.ac.uk/thornton-srv/databases/pdbsum/) indicated that the (CSDIR^913-1190^) region was composed of 18 β-sheets and 2 α-helices Figure 3*F*. Furthermore, the induced fit docking of deltamethrin and malathion compounds with full-length CSDIR protein demonstrated a stable protein-ligand complex, which was then analysed through Molecular Mechanics with Generalised Born and Surface Area solvation (MM-GBSA). The MM-GBSA energy scores and ligand efficiency indicated unfavourable steric interactions and ligand strain during the simulation. The Prime-MMGBSA ligand efficiency of −9.68 and −12.32, were observed for malathion and deltamethrin, respectively, and an MM-GBSA ΔG (binding) −39.61 Kcal/mol and −52.01 Kcal/mol were observed for malathion and deltamethrin, respectively. Additionally, Molecular Dynamics (MD) simulations were performed up to 300 ns for the induced-fit docked ligand-protein complexes for deltamethrin and malathion respectively, Fig. S3 and S5. The Root Mean Square Deviation (RMSD) and Root Mean Square Fluctuation (RMSF) values were calculated for deltamethrin and malathion. The CSDIR-Deltamethrin RMSF graph depicted that the ligand is tightly bound to the active site with an average atomic movement of less than 1.5 Å throughout the MD simulation and the ligand interaction is specific to ligand structure and energetically favourable Fig. S3*A*. The Root means square deviations between the CSDIR’s binding site and bound deltamethrin showed an initial stabilization in the system in the first 50 ns, and the ligand retains most of the docking interactions validating the docking and MM-GBSA calculation. We observed highly stable interaction for the rest of the simulation, though CSDIR protein showed the characteristic hinge movement expanding the active site during 150 to 200 ns simulation time. However, this did not affect the binding of the ligand and some restriction in the movement of protein was seen due to the presence of ligand. Throughout the long simulation, the ligand had not fly off from the initial docking site validating the docking-MM-GBSA studies Fig. S3*C*. Similarly, The CSDIR-Malathion RMSF graph showed that the ligand retained its interacting characteristics while showing strong binding throughout the MD simulation and the ligand interaction is specific to ligand structure and energetically favorable Fig. S5*A*. Additionally, The RMSD graph showed an initial stabilization in the system in the first 50 ns and the ligand retains most of the docking interactions validating the docking and MM-GBSA calculation. The presence of water in the Arg1018 and Glu1021 pockets was found to be most important for stabilizing the molecule. At ∼120 ns, the protein structure movements showed the expansion in the binding pocket with increase in the water molecule weakening the charged interactions between ester moieties and amino acids. Phosphate and ethanol carbons maintained their interactions. This movement of ligand was completely reversed as the pocket reverted to its original shape at ∼200 ns simulation time. This revealed the flexibility of malathion is necessary for ligand binding during changes in the protein hinge movements near the active site. This was followed by similar stabilization as seen before and looks like a cyclic movement. Throughout the long simulation, the ligand had no Flying off from the initial docking site validating the docking-MM-GBSA studies Fig. S5*C*. A detailed schematic representation of amino acid residues and ligand atoms contacts occurring for more than 2% of the simulation time was created for both malathion and deltamethrin (Fig. 3*G*) and (Fig. 3*H*). Crucial amino acid residues that involved in strong molecular interactions with deltamethrin and malathion compounds were identified. For CSDIR-Deltamethrin complex, Trp969 showed aromatic interaction with the benzophenone moiety which is the most crucial for the binding of the ligand. Also, there were key interactions spanning throughout the ligand molecule making it a very stable ligand. The benzophenone moiety was further stabilized by charged and hydrophilic interactions with Arg 1018 and Asp 964. On the other end, the di-halide moiety of deltamethrin remained in a hydrophobic pocket formed by amino acid side chain residues Leu 988, Leu952, and Leu971. The cyanide moiety interacted mainly with Glu1015 and also stabilized by the presence of water molecules which cushion protein movements by changing in numbers with changes in the size of the pocket. While dimethyl-cyclopropane didn’t interact directly with any side chain amino acid residues it provided a rather rigid scaffolding for maintaining the interactions of all the ligand moieties and didn’t disturb the hydrophobic pocket architecture necessary for di-halide moiety binding. Deltamethrin binding had little or no change in the interaction characteristics throughout the long simulation and no fly-off had been observed during the course of the simulation Fig. S4. Similarly, for CSDIR-Malathion complex, Trp969 pi-pi interaction with the phosphorous atom was the most crucial for the binding of the ligand while due to the high number of rotatable bonds (flexibility), there were higher movements in the rest of the molecule but the hydrophobic pocket formed due to Ile1020, Leu952 and Leu986 captured the main aliphatic core and ester sites were stabilized by amphipathic charged pocket mainly comprising of Arg1018 and Glu1021 Fig. S6. Interestingly, Deltamethrin also had the same amino acid (Trp969) as the key interacting partner.

### Biochemical and biophysical characterization of CSDIR protein

**Figure 4:**
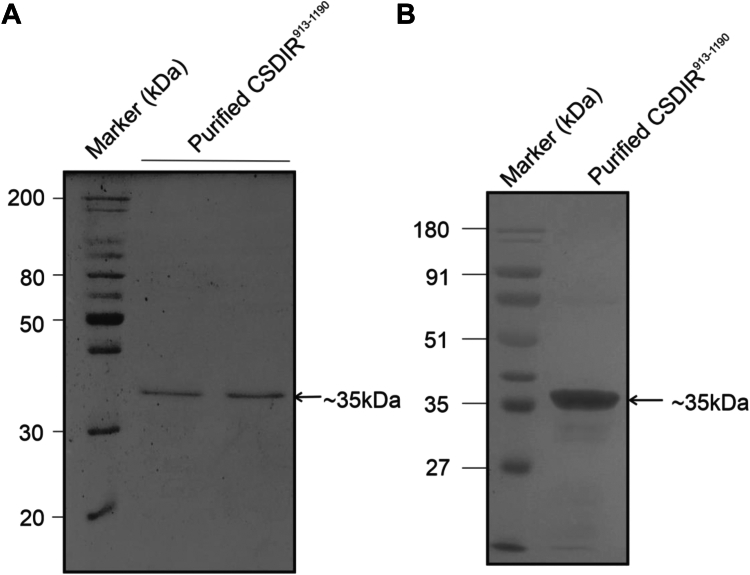
**Recombinant expression and purification of CSDIR in *E. coli*.***A*, SDS-PAGE analysis of recombinant CSDIR^913-1190^ in BL21-DE3 cells of *E. coli. B*, Western blot confirmation of recombinant CSDIR^913-1190^ using Anti-his antibody.

The C-terminal region (CSDIR^913-1190^) was successfully expressed in BL-21(DE3) *Escherichia coli* cells, and the recombinant protein was purified from the soluble fraction using affinity chromatography at an expected size of ∼35 kDa (Fig. 4*A*). Further, CSDIR^913-1190^ protein was confirmed by immunoblotting using polyclonal anti-His antibodies (Fig. 4*B*).

**Figure 5:**
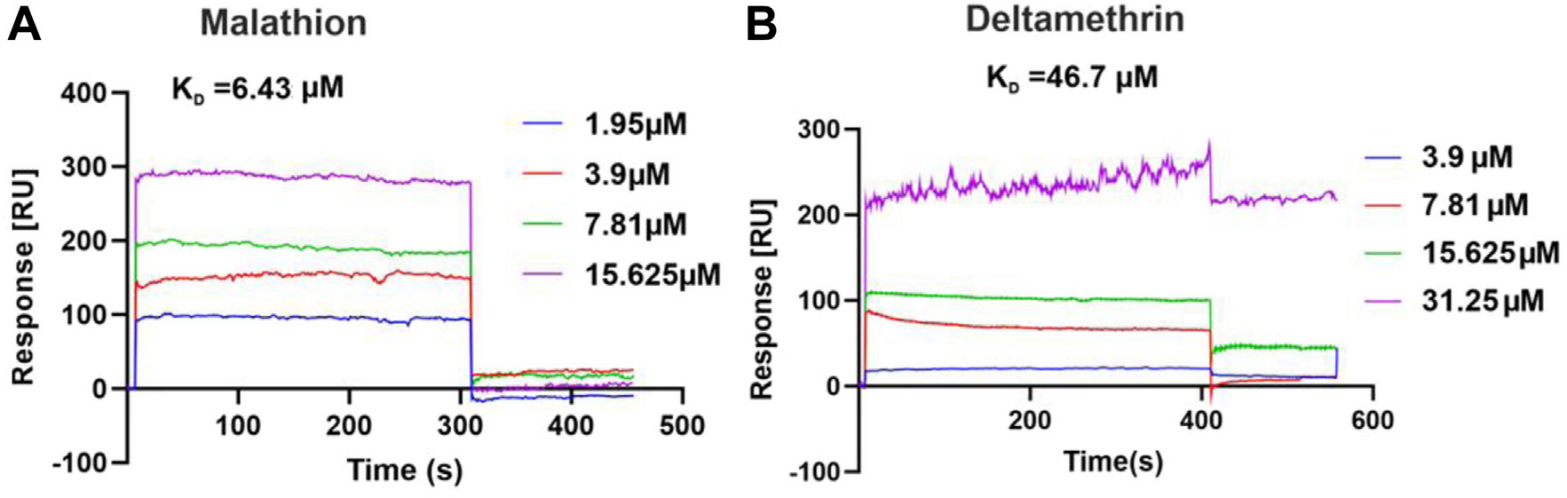
**Molecular Interaction of CSDIR protein with malathion and deltamethrin compounds.***A*, Surface Plasmon Resonance (SPR) showing concentration-dependent binding affinity of recombinant CSDIR^913-1190^ with malathion. *B*, Surface Plasmon Resonance (SPR) showing concentration-dependent binding affinity of recombinant CSDIR^913-1190^ with deltamethrin.

**Figure 6:**
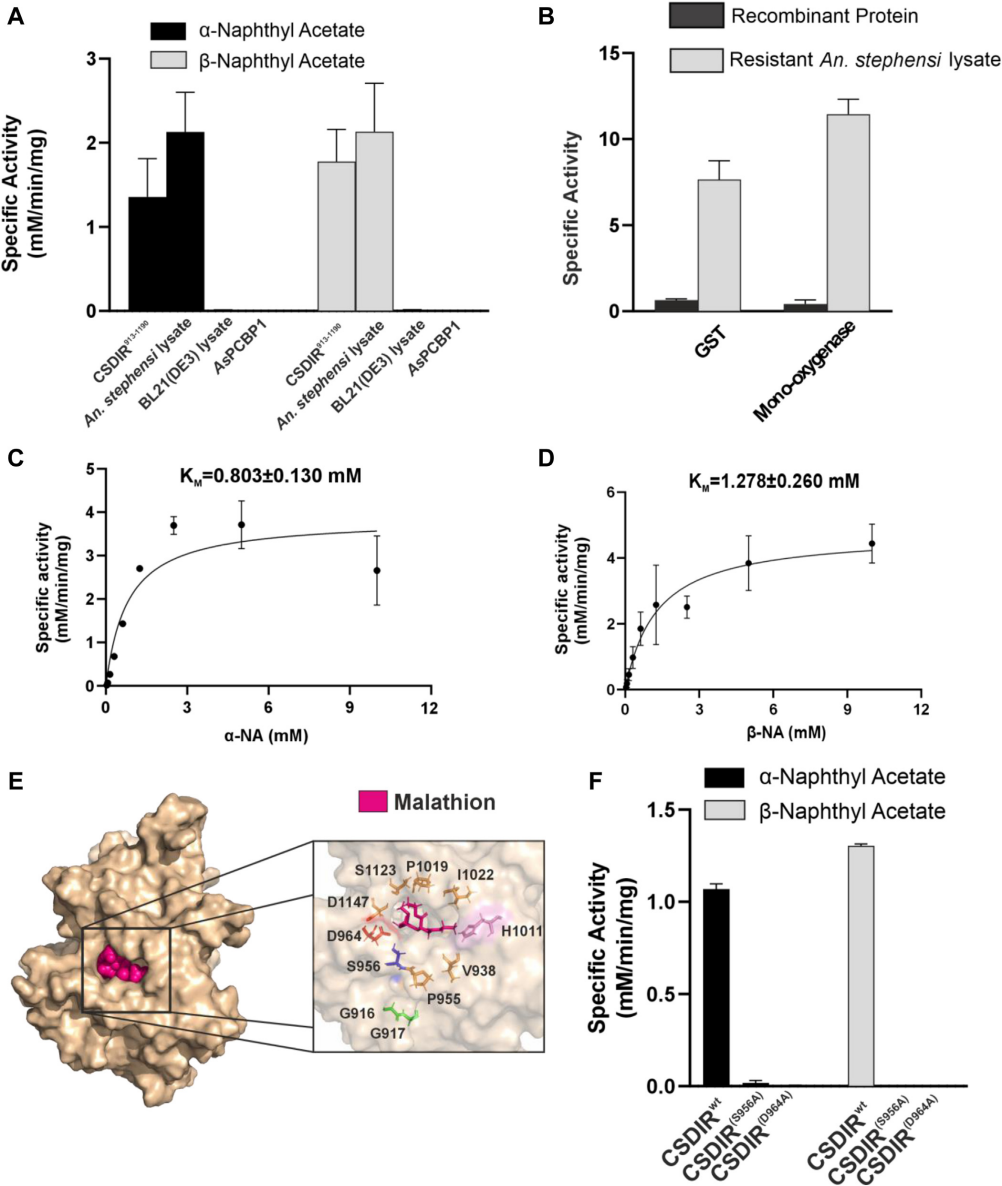
**Recombinant CSDIR**^**913-1190**^**showed esterase-like activity.***A*, the bar graph showing the specific activity of recombinant CSDIR^913-1190^ against α-naphthyl acetate and β-naphthyl acetate compared with resistant female *An. stephensi* lysate. BL21(DE3) lysate and *As*PCBP1 served as a negative control. *B*, the bar graph showing the specific activity of recombinant CSDIR^913-1190^ against GST and Mono-oxygenase compared with resistant female *An. stephensi* lysate. *C*, a graph indicating the binding kinetics of recombinant CSDIR^913-1190^ with α-naphthyl acetate, K_m_ was calculated using non-linear Michaelis-Menten regression. *D*, a graph indicating the binding kinetics of recombinant CSDIR^913-1190^ with β-naphthyl acetate, K_m_ was calculated using non-linear Michaelis-Menten regression. *E*, CSDIR^913-1190^ 3D model showing putative binding pocket. Malathion (as a reference) inside the binding pocket is highlighted in magenta color. Residues S956 (blue), D964(Red), and H1011 (Pink) form the catalytic triad whereas G916 and G917 (green) form the oxyanion hole. *F,* a bar graph showing the specific activity of recombinant CSDIR^913-1190^(CSDIR^wt^) and CSDIR mutants (CSDIR^(S956A)^, CSDIR^(D964)^) against α-naphthyl acetate and β-naphthyl acetate.

**Figure 7:**
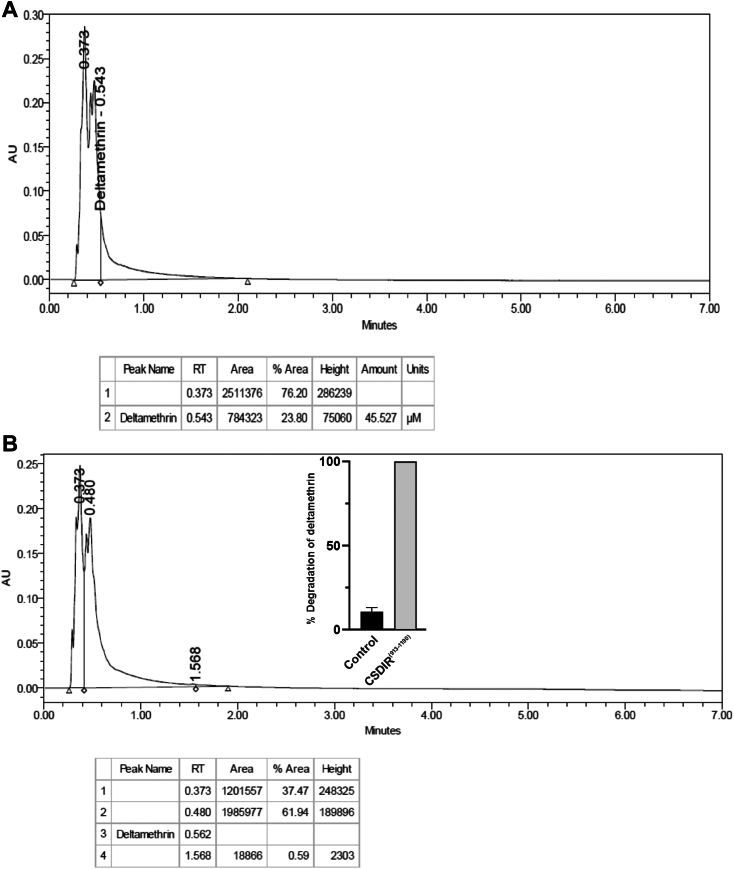
**Deltamethrin degradation by recombinant CSDIR**^**913-1190**^**protein.***A*, UPLC chromatogram showing nonenzymatic control of deltamethrin. *B*, UPLC chromatogram showing degradation of deltamethrin after incubation with CSDIR protein (5 μg). The points above bar shows the standard deviation.

To corroborate the *in silico* findings, we performed Surface Plasmon Resonance (SPR) to validate the biophysical interactions between CSDIR^913-1190^ and deltamethrin/malathion insecticides. The SPR data suggested that CSDIR^913-1190^ indeed had strong binding affinities towards malathion (K_D_ 6.43 μM), and deltamethrin (K_D_ 46.7 μM), respectively (Fig. 5, *A* and *B*). Furthermore, to assesses the enzymatic activity of the CSDIR protein, we performed different detoxification enzymatic assays for-esterase, glutathione-S-transferase (GST), and monooxygenase activities. Interestingly, CSDIR^913-1190^ demonstrated a strong biochemical activity with substrates for esterase activity (α-naphthyl acetate (α-NA)- 1.356 ± 0.262 mM/min/mg and β-naphthyl acetate (β-NA)- 1.777 ± 0.220 mM/min/mg) comparable to the whole *An. stephensi* lysate (α-NA- 2.129 + 0.471 mM/min/mg and β-NA- 2.132 + 0.576 mM/min/mg). The bacterial lysate of *E. coli* BL21-DE3 cell (26) and a recombinant protein *As*PCBP1 (Poly (rC) Binding Protein1)- lacking an esterase activity, served as negative control (Fig. 6*A*). However, negligible activities were observed against GST and monooxygenase substrates (Fig. 6*B*). The CSDIR^913-1190^ showed a higher binding affinity towards α-NA (K_m_ 0.803 ± 0.130 mM) compared to β-NA (K_m_ 1.278 ± 0.260 mM), as shown in Fig. 6, *C* and *D*, respectively, corroborating previous findings (27, 28, 29). These results suggested that the recombinant CSDIR^913-1190^ protein possessed a potent esterase-like activity despite having no conserved sequence similarity with known esterase’s. To exhibit esterase activity, enzymes usually require a catalytic triad formed by nucleophile serine, an acidic residue (aspartic acid/glutamic acid), and histidine. These residues are not contiguous in the primary sequence but are present at a distinct location in the CSDIR protein. Apart from this, they require an oxyanion hole formed by 2 to 3 glycine residues to stabilize the transition during the reaction (30, 31). Using *in silico* analysis, we identified a putative binding pocket, and the presence of residues S956, D964, and H1011 in the close vicinity, that provided strong evidence of forming a catalytic triad, and residues G916 and G917 might be forming an oxyanion hole which could be responsible for the CSDIR^913-1190^ esterase-like activity (Fig. 6*E*). Furthermore, to experimentally validate the role of these crucial amino acid residues in demonstrating esterase activity, we substituted S956, and D964 residues with alanine. Both mutant proteins (CSDIR^(S956A)^, CSDIR^(D964A)^) were successfully purified and confirmed through western blotting (Fig. S8). The purified mutants were tested for esterase activities with (α-NA) and (β-NA). Interestingly, we observed no esterase activities against (α-NA) (CSDIR^S956A^ = 0.018 ± 0.013 mM/min/mg), CSDIR^D964A^ = −0.068 ± 0.073 mM/min/mg) and (β-NA) (CSDIR^S956A^ = −0.146 ± 0.149 mM/min/mg), CSDIR^D964A^ = −0.089 ± 0.040 mM/min/mg) as compared to wild type (CSDIR^913-1190^) (α-NA = 1.069 ± 0.029 mM/min/mg and β-NA = 1.304 ± 0.010 mM/min/mg) (Fig. 6*F*). The data provided concrete evidence that these residues were responsible for the esterase-like activities of the CSDIR^913-1190^ protein. Considerable esterase-like activities and affinities of the CSDIR^913-1190^ protein towards insecticide molecules, supported our hypothesis of the involvement of CSDIR^913-1190^ in mediating insecticide resistance in *An. stephensi*. Additionally, we observed 100% degradation of deltamethrin (50 μM) by 5 μg of CSDIR^913-1190^ protein, compared to nonenzymatic control (10.65 ± 2.47%) using UPLC (Fig. 7). The raw data of the UPLC experiment is provided in the Fig. S7.

### Functional knockdown of the CSDIR protein using dsRNA-mediated gene silencing

**Figure 8:**
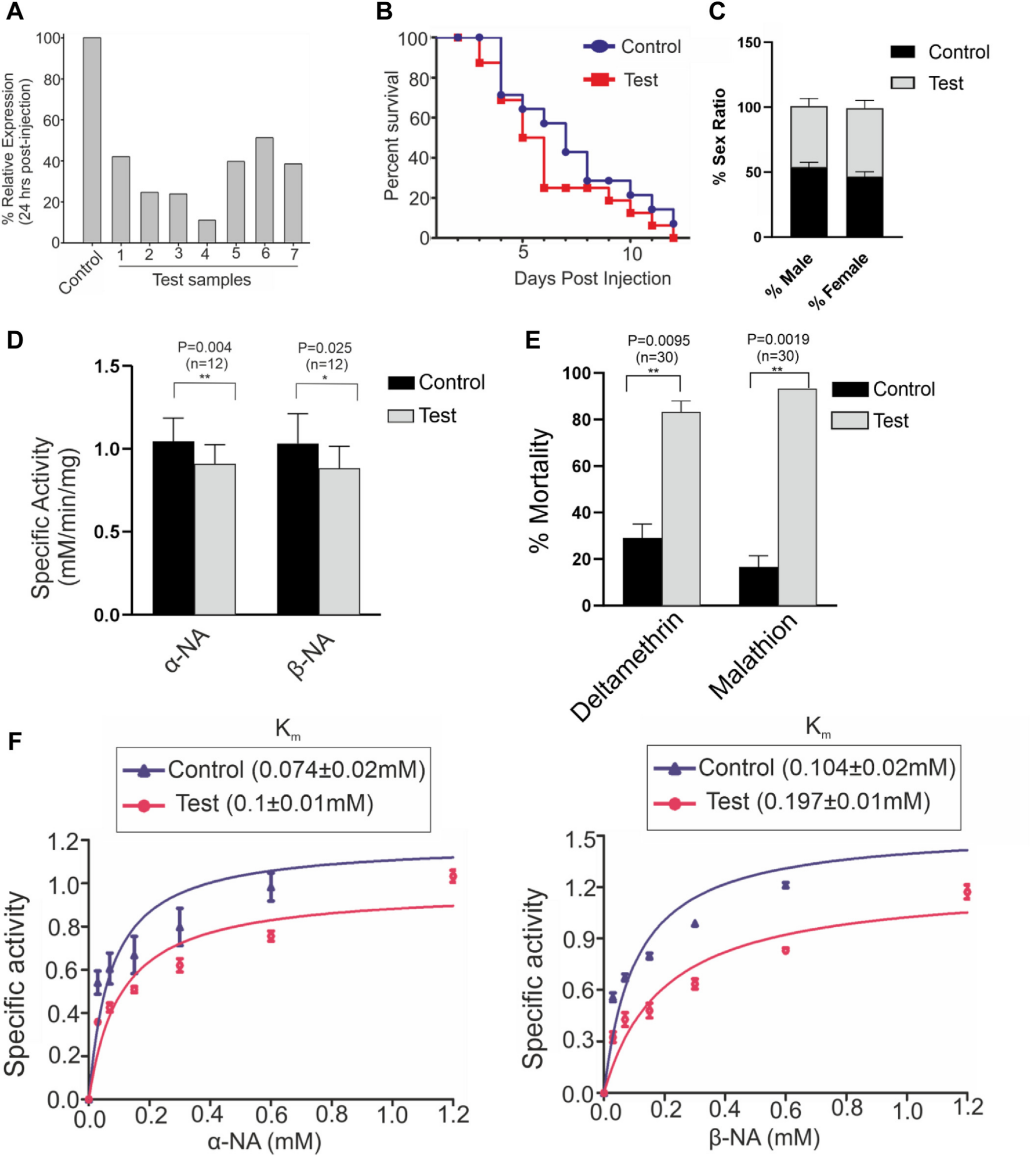
**RNAi-mediated gene knockdown of CSDIR.***A*, percentage relative expression of CSDIR gene after 24 h post-injection, confirming CSDIR gene knockdown, GFP was used as a control (n = 10). Each bar represents an individual mosquito sample (n = 7). *B*, percentage survival curve in control (n = 14) and test (n = 16) group. *C*, a bar graph showing %sex ratio in the control (male(n = 66), female (n = 57)) and test (male (n = 48), female (n = 50)) group depicting CSDIR gene knockdown has not affected mosquito fitness. *D*, a bar graph showing a significant reduction in enzymatic activity against α-naphthyl acetate and β-naphthyl acetate in CSDIR knockdown mosquitoes compared to GFP knockdown mosquitoes. The points above the bar show the standard deviation. Statistical difference was calculated using an unpaired *t* test. *E*, a bar graph showing % mortality after 24 h post-exposure of deltamethrin and malathion in CSDIR and GFP knockdown mosquitoes. The data shows the mean of two biological replicates. Statistical difference was calculated using an unpaired *t* test. *F*, a graph showing an increase in K_m_ value against α-naphthyl acetate and β-naphthyl acetate in CSDIR knockdown mosquitoes compared with GFP knockdown mosquitoes.

A dsRNA-mediated gene silencing experiment was performed to investigate the functional role of the CSDIR gene. Quantitative Reverse Transcriptase- PCR (qRT-PCR) 24 h post-injection demonstrated reduced CSDIR transcripts (∼50%) in the ds*CSDIR*-injected female *An. stephensi* mosquitoes (test) group as compared to ds*GFP-*injected female *An. stephensi* (control) (Fig. 8*A*). The test and control knockdown group of mosquitoes were assessed for fitness parameters such as survival and sex ratio. We observed no significant changes in terms of percent survival in control and test group of mosquitoes. Similarly, no change in control (male 53.64%, female 46.36%) and test (male 47.14%, female 52.85%) groups sex ratio was observed (Fig. 8, *B* and *C*). Furthermore, we found a significant reduction in the esterase activities against α-NA in test group (0.91 ± 0.12 mM/min/mg) compared to the control group (1.04 ± 0.14 mM/min/mg) (*p*-value ≤ 0.004). Similarly, a significant reduction in β-NA activity in the test group (0.88 ± 0.13 mM/min/mg) compared to the control group (1.03 ± 0.18 mM/min/mg) (*p*-value ≤ 0.025) (Fig. 8*D*) was observed. Additionally, we also studied the binding kinetics in the control and test group of mosquitoes with α-NA and β-NA substrates by estimation of Michaelis-Menten constant (K_m_). An increase in the K_m_ value in the test group (0.1 ± 0.01 mM) compared with the control group (0.074 ± 0.02 mM) against α-NA was observed. Similarly, an increase of K_m_ value in the test group (0.197 ± 0.02 mM) compared with the control group (0.104 ± 0.02 mM) against β-NA was observed. The increase in K_m_ values suggested the lowering of affinity of the CSDIR protein towards the esterase substrates in test group compared to control group (Fig. 8*F*). Thereafter, both test and control group mosquitoes were assessed for susceptibility towards deltamethrin and malathion insecticides through WHO adult susceptibility assay, and we found a higher percentage of mortality in the test group (93.30 ± 0.00%) compared to the control group (16.65 ± 3.350%) against malathion. Similarly, a higher percentage of mortality in the test group (83.30 ± 3.30%) compared to the control group (29.15 ± 4.15%) against deltamethrin was also observed (Fig. 8*E*).

## Discussion

The insecticide resistance mechanisms are complex and involve multiple mechanisms such as target-site insensitivity and metabolic resistance through detoxification enzymes. Recently, many novel candidate genes have been shown to be linked with insecticide resistance, however, their functional characterization is still largely unknown (3, 15). In the present study, we identified an uncharacterized CSDIR protein (XP_035915664.1) from deltamethrin and malathion-resistant female *An. stephensi* mosquitoes through differential protein profiling. Computational analysis revealed CUB and Sushi domains at the C-terminal region of the CSDIR protein. These extracellular domains are present in functionally diverse proteins that are involved in cell signalling pathways, complement activation, receptor-mediated endocytosis, tumor suppression, *etc.* The CUB domain is mostly found in developmentally regulated proteins and is known to be involved in drug resistance against chemotherapeutic agents in breast cancer, calcium binding, and peptidase action. CUB domain is also known to be involved in oligomerization and substrate recognition (32, 33, 34). Thus, the presence of the CUB domain in CSDIR protein from deltamethrin and malathion-resistant mosquitoes warrants thorough investigation. The Sushi domain is mostly related to proteins involved in recognition processes such as the complement system (35). The MSA of CSDIR proteins from the major *Anopheles* vectors revealed high conservation of the CUB and Sushi domains; however, we did not find any experimental evidence validating their involvement in the emergence of insecticide resistance in mosquitoes. Our computational and experimental findings suggest a putative mode of action employed by the CSDIR protein. Interestingly, a stable complex formation between the C-terminal CSDIR^913-1190^ protein and ligands-deltamethrin and malathion was observed in MD simulations. Moreover, the residues constituting the putative binding sites for deltamethrin (Trp969, Arg1018, Asp964, Leu988, Leu952, Leu971 and Glu1015) and malathion (Trp969, Ile1020, Leu952, Leu986, Arg1018and Glu1021) were found in the CUB domain of CSDIR protein. However, Trp969 was the most crucial amino acid residue for the binding of both malathion and deltamethrin. The SPR data confirmed a strong interaction between the recombinant CSDIR^913-1190^ protein and ligands- (deltamethrin and malathion- K_D_ 46 μM and 6 μM, respectively). The CSDIR^913-1190^ protein demonstrated a non-canonical esterase activity with (α-NA- 1.356 ± 0.262 mM/min/mg and β-NA- 1.777 ± 0.220 mM/min/mg), comparable to whole mosquito lysate, despite lack of any conserved sequence similarity with known esterase’s. The putative catalytic triad (S956, D964 and H1011) required for the esterase activities was present in the CUB domain; site directed mutagenesis confirmed that S956 and D964 amino acid residues were crucial for the CSDIR’s esterase activity. CSDIR^(S956A)^ and CSDIR^(D964A)^ completely lacked the esterase activities (36, 37). The extracellular nature of the CUB domain and a putative binding site for deltamethrin and malathion implies that in case of resistance development, these insecticide molecules may be metabolized outside of the cell, possibly mitigating the insecticidal action. Our UPLC data clearly indicated that CSDIR protein can directly metabolize deltamethrin. Unfortunately, we could not achieve the metabolic degradation of malathion due to technical difficulties. However, a comprehensive analysis on metabolic degradation of deltamethrin by CSDIR protein should be conducted. Furthermore, the functional knockdown of the CSDIR protein using dsRNA showed ∼50% reduction in CSDIR transcripts, resulting in enhanced deltamethrin and malathion toxicity up to ∼54% and ∼77%, respectively in resistant *An. stephensi* mosquitoes. Additionally, the reduced esterase activity in the resistant mosquito lysate and increased K_m_ values against α-NA and β-NA substrates confirmed the involvement of the CSDIR protein in the detoxification of insecticides. A marginal effect in the K_m_ values of esterase substrates might be attributed to the presence of other esterases in the resistant mosquito lysate. Together, these data identified a novel CSDIR protein with no prior reports of its involvement in insecticide resistance in *Anopheles*. We also provided strong evidence that the CSDIR protein played a key role in mitigating insecticide resistance (organophosphate and pyrethroid) *via* its esterase-like activity. Further studies on the mechanistic aspects of CSDIR protein may enhance our understanding of how extracellular domains can detoxify insecticides outside the cell.

## Conclusion

Altogether, our study identified and characterized a new CSDIR protein in the context of the involvement in the emergence of resistance against multiple insecticides (organophosphates and pyrethroids) in the Indian malaria vector *Anopheles stephensi*. Overall, this study has provided insights into the potential role of CSDIR protein in mitigating insecticidal action. The development of inhibitors to block CSDIR esterase activities needs thorough exploration in the future, as these CSDIR inhibitors might complement the action of currently used insecticides. Ultimately, the study will aid in the development of new adjunct insecticidal molecules, and further prolong the delay of the emergence of insecticide resistance for our current vector control strategies.

## Experimental procedures

### Comparative proteomics of insecticide-resistant/susceptible field-collected *A. stephensi*

Adult mosquitoes were reared at (27 ± 2 °C) and 70% humidity with a constant 12 h day/night cycle and fed on fed on 6% glucose soaked in cotton pads. The deltamethrin and malathion resistance status of the *An. stephensi* was determined using the WHO adult susceptibility assay (38). Deltamethrin (0.05%) and malathion (5%) impregnated papers were obtained from the Vector Control Research Unit (VCRU), University Sains Malaysia, Malaysia (http://www.usm.my). Fully fed mosquitoes were exposed to deltamethrin and malathion-impregnated papers for 1 h in 3 to 5 replicates with appropriate controls. After exposure, mosquitoes were transferred to a holding tube containing a glucose pad, moistened from the bottom to maintain relative humidity (70–80%) and temperature (27 ± 2 °C). Percentage mortality was calculated after 24 h (39). The susceptibility towards deltamethrin and malathion was assessed as per standard WHO criteria. The mosquitoes with 98 to 100% mortality considered as susceptible, below 90% mortality considered as resistant (40). The deltamethrin/malathion-resistant and susceptible mosquitoes were separated for lysate preparation and protein profiling. Briefly, individual female mosquitoes were homogenized in 50 to 100 μl of ice-cold 1× PBS using a hand homogenizer. The clear supernatant was obtained by high-speed centrifugation (14,000 rpm, 4 °C for 15 min). Comparable sample lysates from resistant and susceptible mosquitoes were analyzed on 12% SDS-PAGE followed by silver staining as per the manufacturer’s protocol (Focus Fast Silver, G-biosciences). Differentially expressed protein bands were selected and sent for LC/MS-MS at the Central Instrumentation Facility, University of Delhi, India. A nano HPLC (Thermo Scientific Easy-nLC 1200) coupled with a Thermo Scientific Q Exactive Orbitrap mass spectrophotometer was used for the identification of proteins. ∼2 μg peptide was loaded with a flow rate of 300 nl/min on the analytical column (PepMap RSLC C18 2 μm, 75 μm × 50 cm (Thermo Scientific). The mobile phase for the Liquid chromatography was as follows: water/acetonitrile/formic acid (A, 98/2/0.1%; B, 20/80/0.1%). The analytical separation was achieved with the following gradient conditions: initial 5% B for 2 min, followed by a liner gradient 5%-45%B in 102 min. Finally, the gradient was increased up to 90%B in 1 min and remained stable for up to 10 min. The initial chromatography conditions were restored in 1 min and maintained for 4 min. The MS1 was operated on resolution of 70,000 with a mass range of 350 to 2000 m/z. The AGC was set to 3e^6^ and a maximum ion transfer time of 50 ms. The resolution for the MS-MS(MS2) was set to 17,500 with a mass range of 200-2000 m/z. The AGC was set to 1e^5^ and a maximum ion transfer time of 120 ms. The normalised collision energy was set to 27 and a dynamic exclusion time of 50s. Proteome Discoverer 2.4 software was used for protein sequence identification and UniProt database (https://www.uniprot.org/) was used for the validation of identified protein sequence. Detailed methodology is provided in the Supplementary information.

### Computational analysis of XP_035915664.1 (uncharacterized protein)

Out of the three distinctly expressed protein bands from resistant and susceptible *An. stephensi* mosquitoes, protein XP_035915664.1 from band *c* was chosen for further investigation (Table 1). The XP_035915664.1 orthologue sequences were retrieved from NCBI and Clustal Omega was used to create Multiple Sequence Alignment (MSA). The maximum likelihood tree for paralogs and orthologs was constructed using MEGA11 (41). The Jones-Taylor-Thornton (JTT) model with 1000 bootstrap replicates was used for the construction of the tree. The nonsynonymous to synonymous substitution ratios (Ka/Ks) were calculated using the PAML 4 (42). The orthologs of the clustered genes were downloaded from VectorBase in 7 *Anopheline* and 2 *Culicine* species. The Ka/Ks ratios were used to assess the selection pressure on XP_035915664.1 and Ka/Ks ratio > 1, < 1, or = 1 indicated positive, negative, or neutral evolution, respectively. Additionally, the site-specific positive selection and purifying selection were assessed by using the SELECTON server (43). The model structure of XP_035915664.1 protein was generated using the I-TASSER server (https://zhanggroup.org/I-TASSER/) and the active site was mapped through COACH analysis (https://seq2fun.dcmb.med.umich.edu/COACH/). For the presence of CUB & Sushi domains and its role in mediating insecticide resistance, we shall identify XP_035915664.1 protein as CUB and Sushi Domain Containing Insecticide Resistance protein (CSDIR protein). The validation of protein structure was performed through CASP-13 accredited online servers (https://predictioncenter.org/casp13/). Significantly higher expression of band *c* (Fig. 1) containing the CSDIR protein in resistant *An. stephensi* prompted us to perform molecular docking with deltamethrin and malathion as ligands, which was performed through the PatchDock server (http://bioinfo3d.cs.tau.ac.il/PatchDock/) followed by FireDock refinement (http://bioinfo3d.cs.tau.ac.il/FireDock/). The 2D and 3D structure of deltamethrin and malathion ligands were retrieved from the NCBI PubChem database. The ligand structures were generated using the LigPrep application in the Schrödinger suite with energy optimization and minimization. To perform flexible protein-ligand docking the Induced Fit Docking (IFD) application in the Schrodinger suite was used. The energy scoring was performed through extra precision (XP) within 30.0 kcal/mol of the best structures, comprising 20 structures utilizing the generated IFD (kcal/mol) and Glide score (kcal/mol). The Glide score comprised various energies involved in binding site and ligand interactions whereas the IFD score is generally calculated by adding prime energy calculations. The Prime-MM-GBSA score which calculates additional ligand strain in the predicted docked pose was used to select the top conformer. To evaluate the binding stability of CSDIR protein with deltamethrin and malathion, a 300 ns long Molecular Dynamics (MD) simulation was performed using the Desmond module of the Schrödinger software in the presence of water and physiological ions. A 10 Å × 10 Å x 10 Å orthorhombic box around the protein-ligand complex was used to determine the boundary conditions. The system was then solvated in a predefined TIP3P water arrangement. The protein model was neutralized with access to Na^+^/Cl^-^ ions with an excess 0.15 NaCl simulating physiological conditions. Model system relaxations were performed before running simulations in the NPγT ensemble class at constant temperature (300.0 K), pressure (1.013 bar), and surface tension (0.0 bar Å). Thereafter, energy was recorded at regular intervals of 1.2 ps for 100 ns simulation time with a 20 ps trajectory recording (5 K frames). Additionally, a simulation interaction diagram (SID) was generated using trajectory analysis (44).

### Heterologous production of CSDIR^913-1190^ protein and mutants

The gene sequence of the CSDIR protein was retrieved from the National Center for Biotechnology Information (NCBI). The C-terminal region (CSDIR^913-1190^) was selected due to the presence of CUB and Sushi domains and a putative ligand binding pocket as indicated in our *in silico* analysis and enzymatic assays as described later in the methods section. A synthetic construct of the C-terminal region (CSDIR^913-1190^) ligated in the pET28a vector was obtained from Eurofins Genomics, India. The construct was transformed into *E. coli* BL21(DE3) cells. The expression was optimized in LB media containing kanamycin (50 μg/ml), followed by induction with 0.5 mM IPTG at 16 °C overnight. Cells were harvested and resuspended in the buffer containing 50 mM Tris, 500 mM NaCl, 5 mM β-mercaptoethanol, 0.1% Triton X-100, and 5% glycerol. Cells were lysed by sonication for 30 to 40 min, 15 s on and 30 s off at 60% pulse rate, followed by centrifugation at 13,000 rpm at 4 °C for 20 min. The soluble fraction was selected for recombinant protein purification of the CSDIR protein. Proteins were purified using Ni-NTA affinity chromatography and analyzed by 12% SDS-PAGE. To validate the involvement of crucial amino acids S956, D964 and H1011 in exhibiting esterase activity, we created single point mutation by substituting residues with Alanine. The site directed mutagenesis was performed using NEB Q5-Site Directed Mutagenesis kit as per kit protocol. Wild Type CSDIR construct was used as the template. The primers used for mutagenesis has been shown in the Table S1. The mutations were confirmed using DNA sequencing carried out by Eurofins Genomics, India. CSDIR^(S956A)^, CSDIR^(D964A)^, mutant recombinant proteins were purified from the soluble fraction as per CSDIR^wt^ protocol. Unfortunately, we were not able to express H1011A mutant in BL21(DE3) cells. For immunoblotting, SDS-PAGE resolved samples were transferred onto a nitrocellulose membrane (Bio-Rad), further blocked with 5% BSA, overnight at 4 °C. Anti-His HRP-conjugated antibodies (Sigma Aldrich) diluted (1:3000) were used to probe the purified proteins. The blot was developed using 3,3′-Diaminobenzidine (DAB) (Sigma Aldrich) and 0.035% hydrogen peroxide (H_2_O_2_).

### Enzymatic activity assays of CSDIR protein

To ascertain any kind of enzymatic activity conferred by the CSDIR protein, we performed-esterase, monooxygenase, and glutathione-S-transferase enzymatic assays using CSDIR^913-1190^ recombinant protein. Until specified, all assays were performed in three biological replicates, and data were presented as mean ± SD. All the graphs were plotted using GraphPad Prism software.

#### Esterase assay

Briefly, 200 μl of α-naphthyl acetate (α-NA) (from 0.3 mM α-NA in 0.02 M phosphate buffer, pH 7.2) and 200 μl of β-naphthyl acetate (β-NA) (from 0.3 mM β-NA in 0.02 M phosphate buffer, pH 7.2) was added to wells containing the 10 μl CSDIR^913-1190^ protein and mosquito lysate (39, 45). The reaction was incubated for 15 min and terminated by adding 50 μl of O-Dianisidine stain solution (22.5 mg O-Dianisidine in 2.25 ml distilled water, and 5.25 ml of 5% sodium lauryl sulfate diluted in 0.1 M phosphate buffer, pH 7.0). The control well contained 10 μl of distilled water 200 μl of α/β-NA reagent and 50 μl of O-Dianisidin stain. The BL21(DE3) cells with no plasmid were induced similarly as CSDIR protein and soluble fraction were used as a negative control in the assay. Additionally, in-house generated non-esterase recombinant *Anopheles stephensi* Poly (rC)-Binding Protein1 (*As*PCBP1) also served as negative control in the assay. Enzyme activity reads were taken at 570 nm as an endpoint. The results were presented as mM/min/mg protein. Protein estimation was performed using the Bradford protein estimation assay.

#### Glutathione S-transferase assay

Briefly, 50 μl of reduced glutathione (GSH) solution (from 1.9 mM GSH in 0.05 M phosphate buffer (pH 6.8) and 50 μl of 1-chloro 2,4 dinitrobenzene (CDNB) solution (0.01 gm CDNB in 0.5 ml acetone and 50 ml of 0.05 M phosphate buffer) was added to wells containing 20 μl of CSDIR^913-1190^ protein and mosquito lysate. The control well has 20 μl of distilled water 50 μl of GSH and 50 μl CDNB solutions. The enzyme activity was measured at 340 nm continuously for 5 min. The GST-specific activity was represented as μmol/min/mg protein (46). The experiment was performed in two biological replicates, and data were presented as mean ± SD.

#### Monooxygenase assay

To assess the monooxygenase activity, the estimation of heme content in non-blood-fed mosquitoes correlated with cytochrome P450 (CYP_450_) activity, because most of the heme is associated with CYP_450_ in mosquitoes (47). 20 μl of mosquito lysate and CSDIR^913-1190^ protein was added to the wells, followed by 80 μl of 0.0625 M potassium phosphate buffer (pH 7.2). Thereafter, 200 μl of 3,3,5,5′-tetramethyl benzidine (TMBZ) (0.01gm TMBZ dissolved in 5 ml methanol and mixed with 15 ml of 0.25 M sodium acetate buffer pH 5.0). 20 μl of 3% H_2_O_2_ was added to the reaction, followed by incubation for 10 min. Enzyme activity was measured at 650 nm. CYP_450_ specific activity was represented as μmol/min/mg protein (48). The experiments were performed in two biological replicates, and data were presented as mean ± SD.

#### Ultra Performance Liquid chromatography (UPLC)

To investigate the insecticidal molecules (Deltamethrin) degradation by CSDIR recombinant protein, we performed UPLC at Central Instrumentation Facility, University of Delhi, India. Serial dilution of Deltamethrin ranging from 500 μM to 31.3 μM was prepared in methanol for creating an analytical standard. 50 μM Deltamethrin was preincubated in 250 μl Tris-NaCl buffer (50 mM Tris, 50 mM NaCl) pH-7.4 before adding CSDIR protein’s concentration of 5 μg in the reaction. Tris-NaCl buffer with 50 μM deltamethrin was served as a negative control. The reaction was incubated at 30 °C for 1 h. The reaction was finally quenched with the addition of 250 μl chilled methanol (49, 50, 51). The samples were centrifuged and filtered through 0.22 μm filter before loading on the Waters Acquity UPLC instrument. The samples were allowed to run on the Waters BEH C18 (50 mm∗2.1 mm), particle size 1,7 μm column at 30 °C with Isocratic flow 10:90 (water: Acetonitrile). The samples with 5 μl injection volume were allowed to run at a flow rate of 0.4 ml/min for 7 min. The peaked were detected by PDA eλ Detector at 232 nm wavelength. The experiment was performed with two biological replicates.

### Biophysical interaction of CSDIR^913-1190^ protein with ligands-deltamethrin and malathion

To experimentally validate the molecular interactions of recombinant CSDIR^913-1190^ protein with deltamethrin and malathion insecticides, surface plasmon resonance (SPR) experiments were carried out using Auto ESPRIT instrument at Advanced Instrumentation Research Facility, Jawaharlal Nehru University, New Delhi. The SPR gold chip was activated by 0.2 M N-ethyl-N0-(dimethylamino-propyl) carbodiimide (EDC) and 0.05 M N-hydroxysuccinimide (NHS) (Sigma-Aldrich) solution. CSDIR^913-1190^ protein was immobilized on the SPR sensor chip through covalent amine coupling. Different concentrations of deltamethrin and malathion were injected at a constant flow rate of 50 μl/min to study the interaction kinetics. 20 mM NaH_2_PO_4_, 150 mM NaCl, and 5 mM EDTA pH 8.0 were used for immobilization and binding solutions. 50 mM NaOH was used for the regeneration of the sensor chip. Analysis and calculations were carried out in Autolab ESPRIT instrument’s kinetic evaluation software and graphs were made using GraphPad Prism. The data were fit with a steady-state model to obtain the K_D_ (equilibrium dissociation constant).

### Targeting CSDIR protein using dsRNA-mediated gene silencing

Functional knockdown of the gene expressing CSDIR protein was achieved through RNAi-mediated gene silencing. 500 bp dsRNA template was designed from *An. stephensi* cDNA with the dsRNA forward primers and reverse containing T7 promoter site (Table S1). The purified PCR product was processed for dsRNA synthesis with a MEGASCRIPT transcription kit (Invitrogen) as per the manufacturer’s protocol. Approximately, 300 nl of purified dsRNA was injected into the thorax of 3-day-old deltamethrin and malathion-resistant *An. stephensi* female mosquitoes by a Pneumatic Picopump PV820 (World Precision Instrument Inc). Non-endogenous GFP dsRNA was injected at the same concentration, serving as a control. dsRNA-injected mosquitoes were allowed to recover at RT for 1 h, followed by rearing in normal conditions (15). Silencing was confirmed through qRT-PCR after 24 h post-injection. RNA was isolated using the Trizol (Invitrogen, California) method. First-strand cDNA was synthesized using a Promega cDNA synthesis Kit (Thermo Scientific). CFX96 Real-Time PCR machine (Bio-Rad) was used to assess the gene expression by SYBR green qPCR master mix (Thermo Scientific). The actin (ASTEI20_042454) gene of *Anopheles stephensi* was used as a normalizing control, and the primer sequence is provided in Table S1. Cycle Threshold (CT) values were used for the quantitative analysis during the product amplification phase. Finally, relative quantifications were calculated by using, the 2ˆ^-ΔΔCT^ method. The primer sequences have been provided in Table S1. qRT-PCR was performed with the following conditions: 95 °C- 5 min, 95 °C-30 s, 56 °C- 45 s, repeated for 39 cycles.

## Data availability

All data described are contained within the manuscript or available as supporting information.

## Supporting information

This article contains supporting information.

## Conflict of interest

The authors declare that they have no conflicts of interest with the contents of this article.
