# Supplementary material for: A cub and sushi domain-containing protein with esterase-like activity confers insecticide resistance in the Indian malaria vector Anopheles stephensi
Source: J Biol Chem. 2024 Sep 10;300(10):107759. doi: 10.1016/j.jbc.2024.107759 (PMC11474193; doi:10.1016/j.jbc.2024.107759)
Supplement: Supplementary Information [file mmc1.docx]

# SUPPLEMENTARY INFORMATION

**Table 1.**

| **PRIMER NAME** | **SEQUENCE** |
| --- | --- |
| CSDIR qRT  Forward | ACGCTCGAGCACCTGATAGT |
| CSDIR qRT  Reverse | TCCATCGAGTTGGAGAATCC |
| CSDIR dsRNA  forward | TAATACGACTCACTATAGGGTTCTGTCGTCCGTACCGGTCGGAT |
| CSDIR dsRNA  Reverse | TAATACGACTCACTATAGGGACGTCGTCGTGTCCTTCATACAG |
| Actin Forward | TGCGTGACATCAAGGAGAAG |
| Actin Reverse | GATTCCATACCCAGGAACGA |
| CSDIR^(S956A)^ Forward | GCTTTCTGATGACCCCGGCGTATCCG |
| CSDIR^(S956A)^ Reverse | CCTGGCGCAGCGGATGTTTCAG |
| CSDIR^(D964A)^ | GAAATATTATATTGGCGCGAGCACCTG |
| CSDIR^(D964A)^ | GGATAGCTCGGGGTCATCAGAAAG |
| CSDIR^(H1011A)^ Forward | GTTTGCGAGTTGTACCGAAG |
| CSDIR^(H1011A)^ Reverse | AGGGTCTGATTGGTATTCAG |

**Figure 1. Adult susceptibility assay of deltamethrin and malathion resistant and susceptible *Anopheles stephensi* lines.** A bar graph showing % mortality *As*Chennai (n=100) and *As*Mewat (n=100) after deltamethrin and malathion exposure to *Anopheles stephensi* female mosquitoes. The points above bars shows standard deviation.


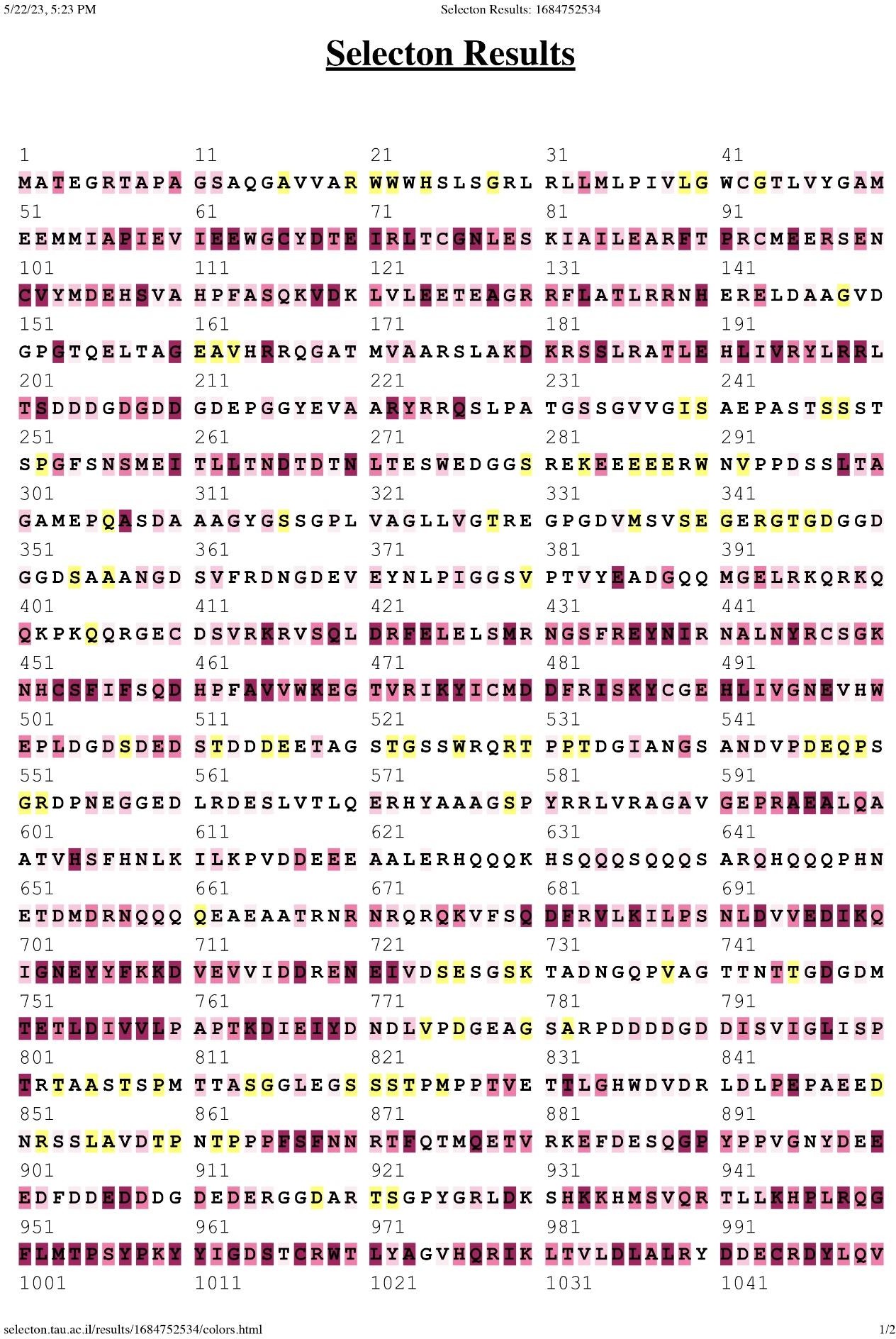


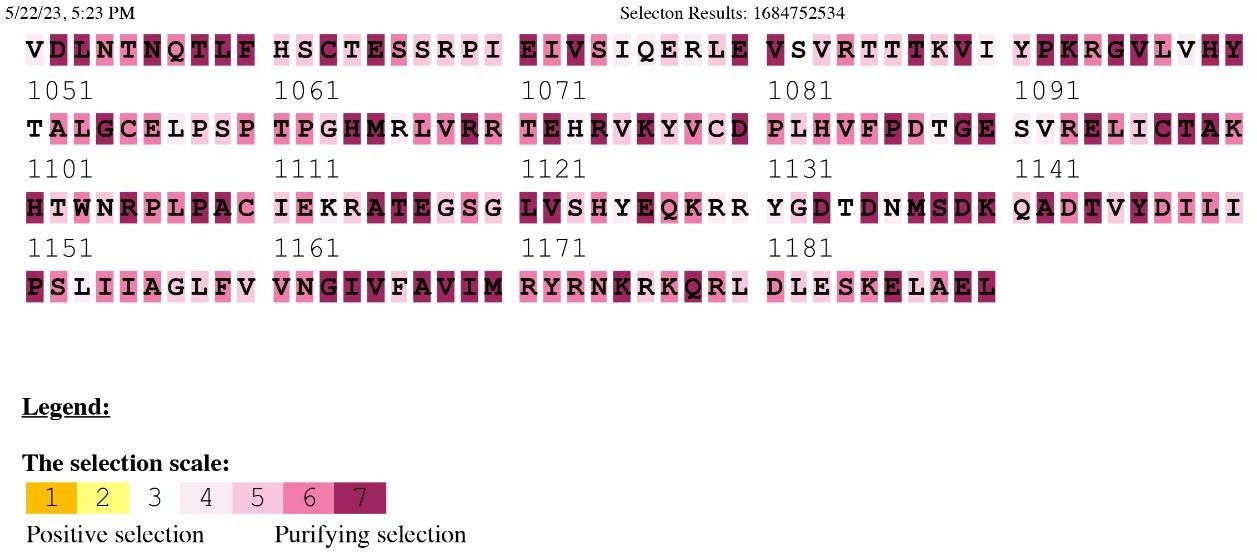


# Figure 2. Nonsynonymous/substitution mutation (Ka) and synonymous/ silent mutation (Ks) rates in the CSDIR protein sequence.


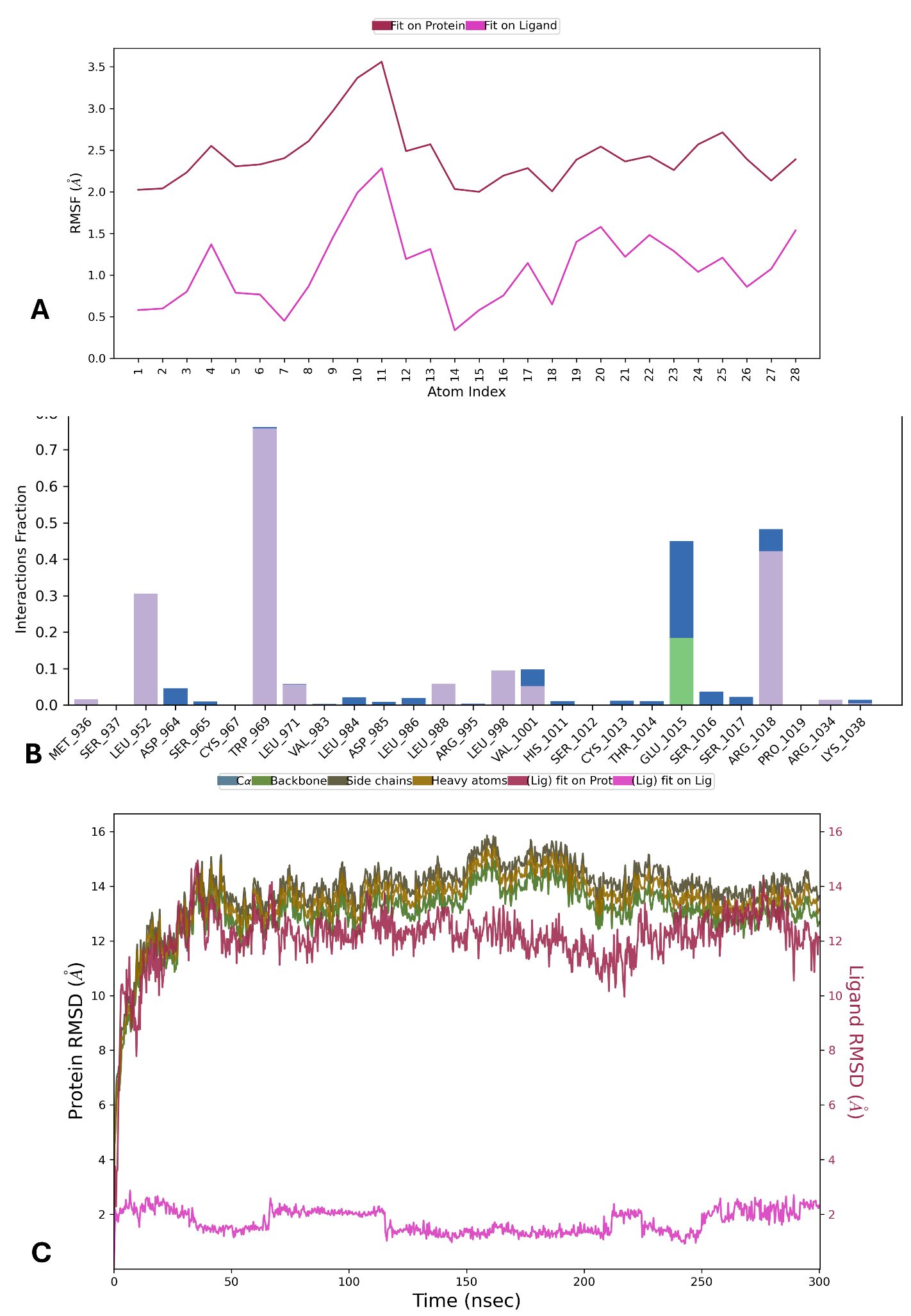


**Figure 3. Results of MD simulation between CSDIR and Deltamethrin.** (A) RMSF fitting graph for all the atoms of deltamethrin during the 300ns MD simulation. The Y-axis shows the distance between the ligand and binding site in Angstrom and the X-axis is the atom numbering according to IUPAC ignoring hydrogen. (B) Bar graph showing CSDIR amino acid residues that are interacting with the ligand during the entire simulation. (C) Root Means Square Deviations (RMSD) between the CSDIR binding site and bound deltamethrin. The left Y-axis shows protein RMSD indicating the evolution of protein during the simulation whereas the right Y-axis indicates ligand RMSD indicating the evolution of ligand during the simulation.


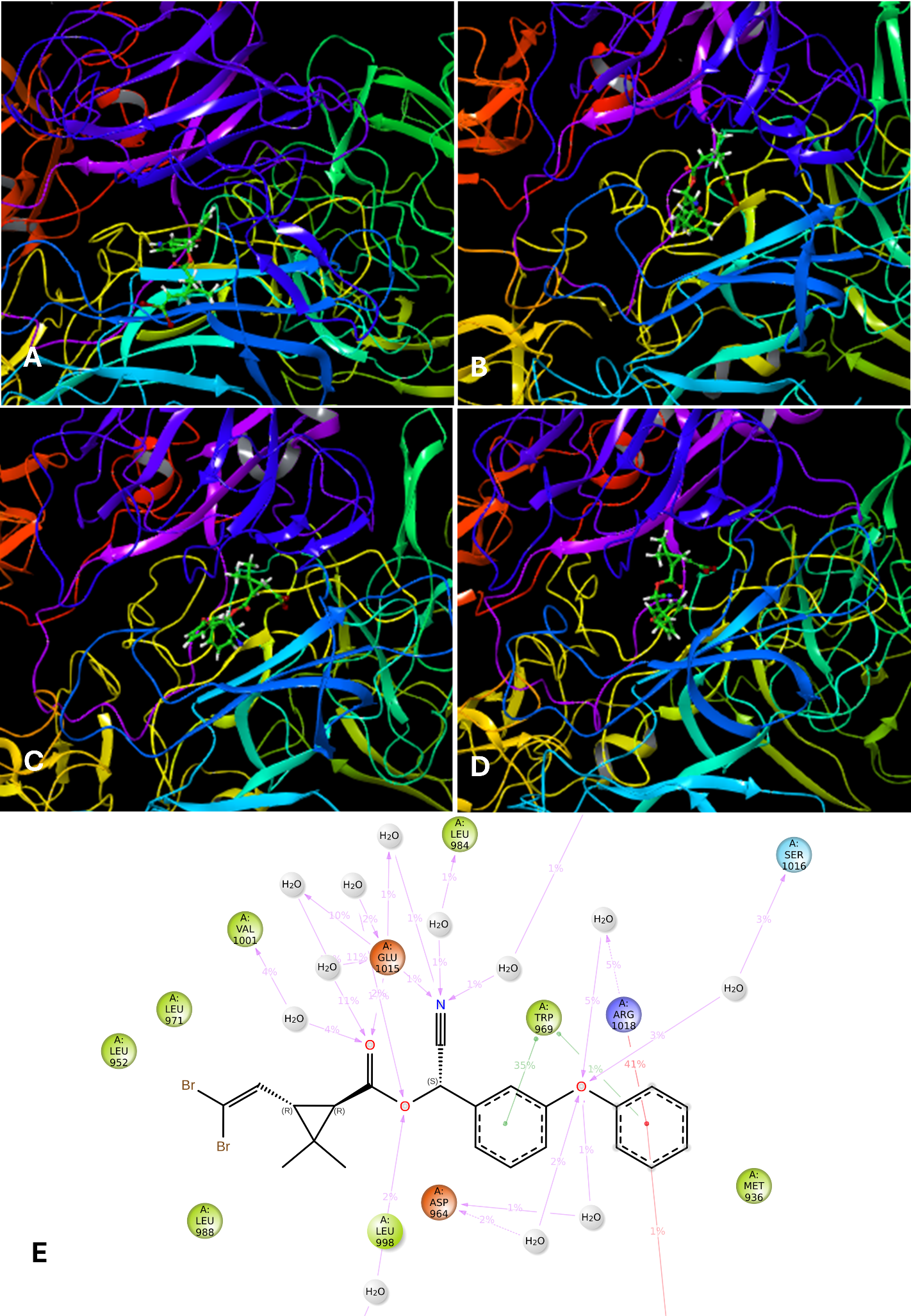


**Figure 4. MD simulation interactions between CSDIR and Deltamethrin.** 3D interaction schematic representation of Ligand interaction at (A). 0ns (original docking). (B). 100ns (C). 200ns (D). 300ns. The ligand (licorice model) was tightly bound to the binding pocket throughout the simulation without flying off. The movements in the ligand complemented the movements in the protein backbone (ribbon cartoon) (E) A schematic of detailed ligand atom interactions with the protein residues. Interactions that occur more than 2.0% of the simulation time in the selected trajectory (0.00 through 300.00 ns), are shown.


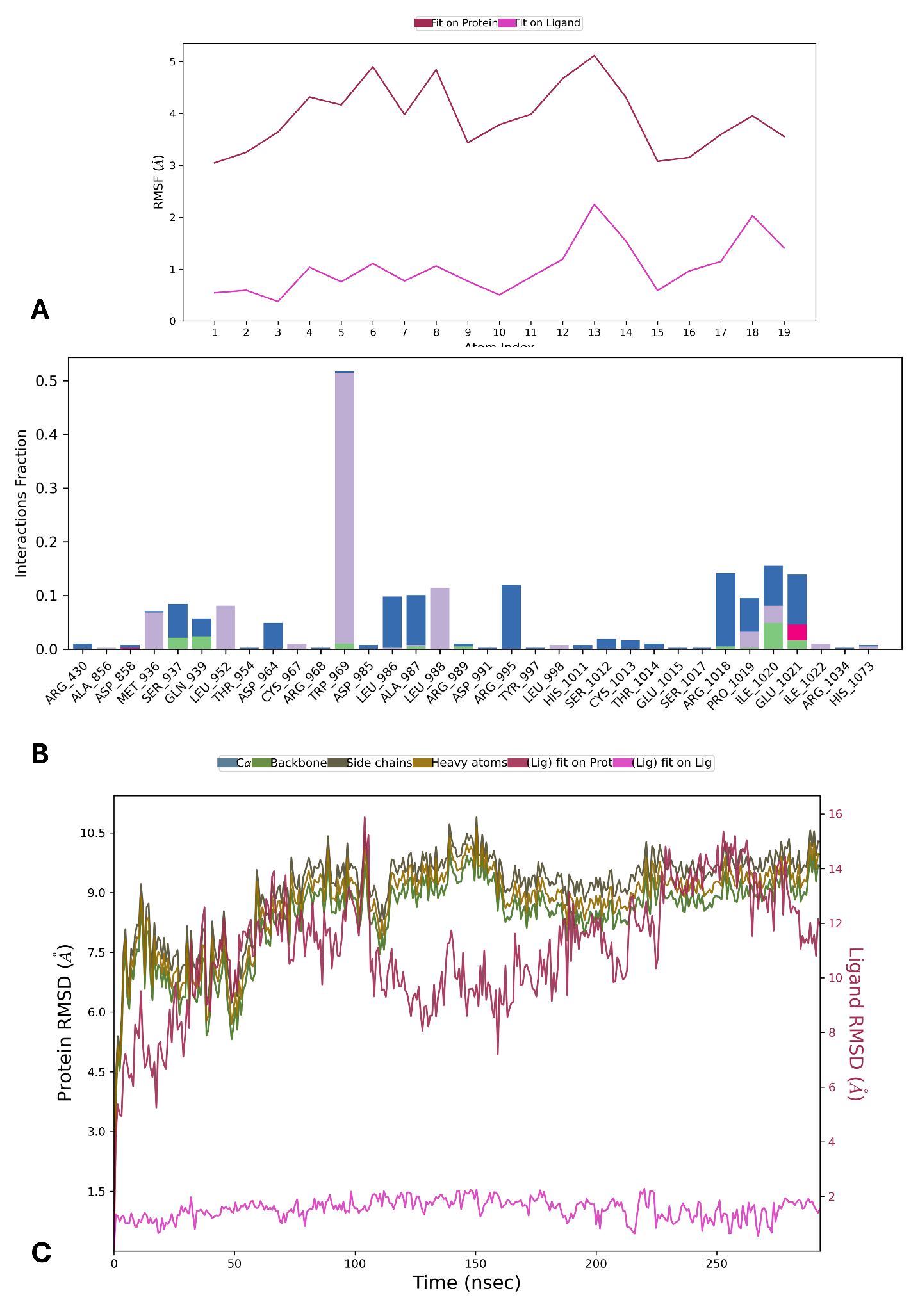


**Figure 5. Results of MD simulation between CSDIR and Malathion.** (A) RMSF fitting graph for all the atoms of malathion during the 300ns MD simulation. The Y-axis shows the distance between the ligand and binding site in Angstrom and the X-axis is the atom numbering according to IUPAC ignoring hydrogens. (B) Bar graph showing CSDIR amino acid residues that are interacting with the ligand during the entire simulation. (C) Root Mean Square Deviations (RMSD) between the CSDIR binding site and bound malathion. The left Y-axis shows protein RMSD indicating the evolution of protein during the simulation whereas the right Y-axis indicates ligand RMSD indicating the evolution of ligand during the simulation.


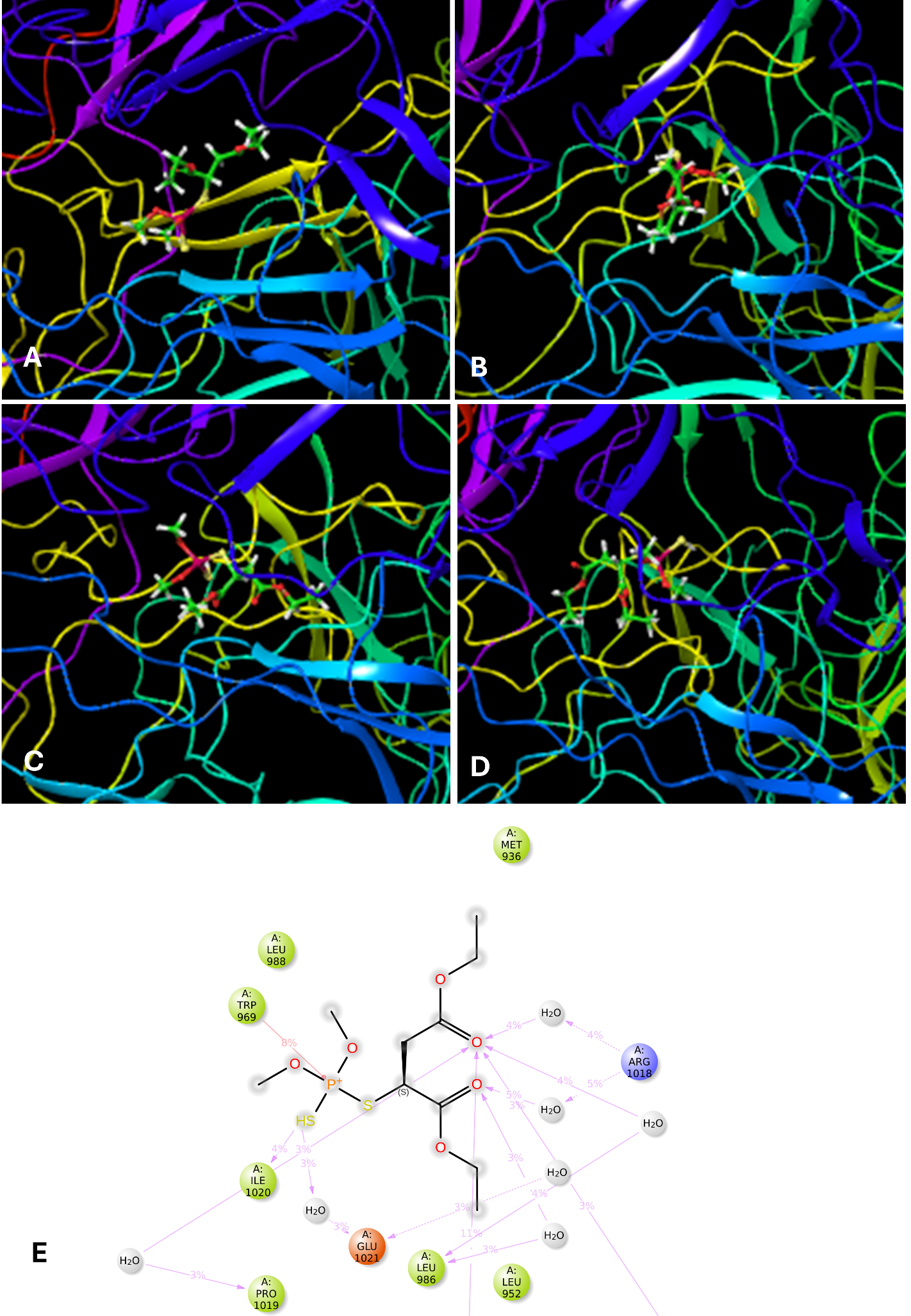


**Figure 6. MD simulation interactions between CSDIR and Malathion.** 3D interaction schematic representation of Ligand interaction at (A). 0ns (original docking). (B). 100ns (C). 200ns (D). 300ns. The ligand (licorice model) stayed in the binding pocket throughout the simulation without flying off. The movements in the ligand complemented the movements in the protein backbone (ribbon cartoon) (E) A schematic of detailed ligand atom interactions with the protein residues. Interactions that occur more than 2.0% of the simulation time in the selected trajectory (0.00 through 300.00 ns) are shown.


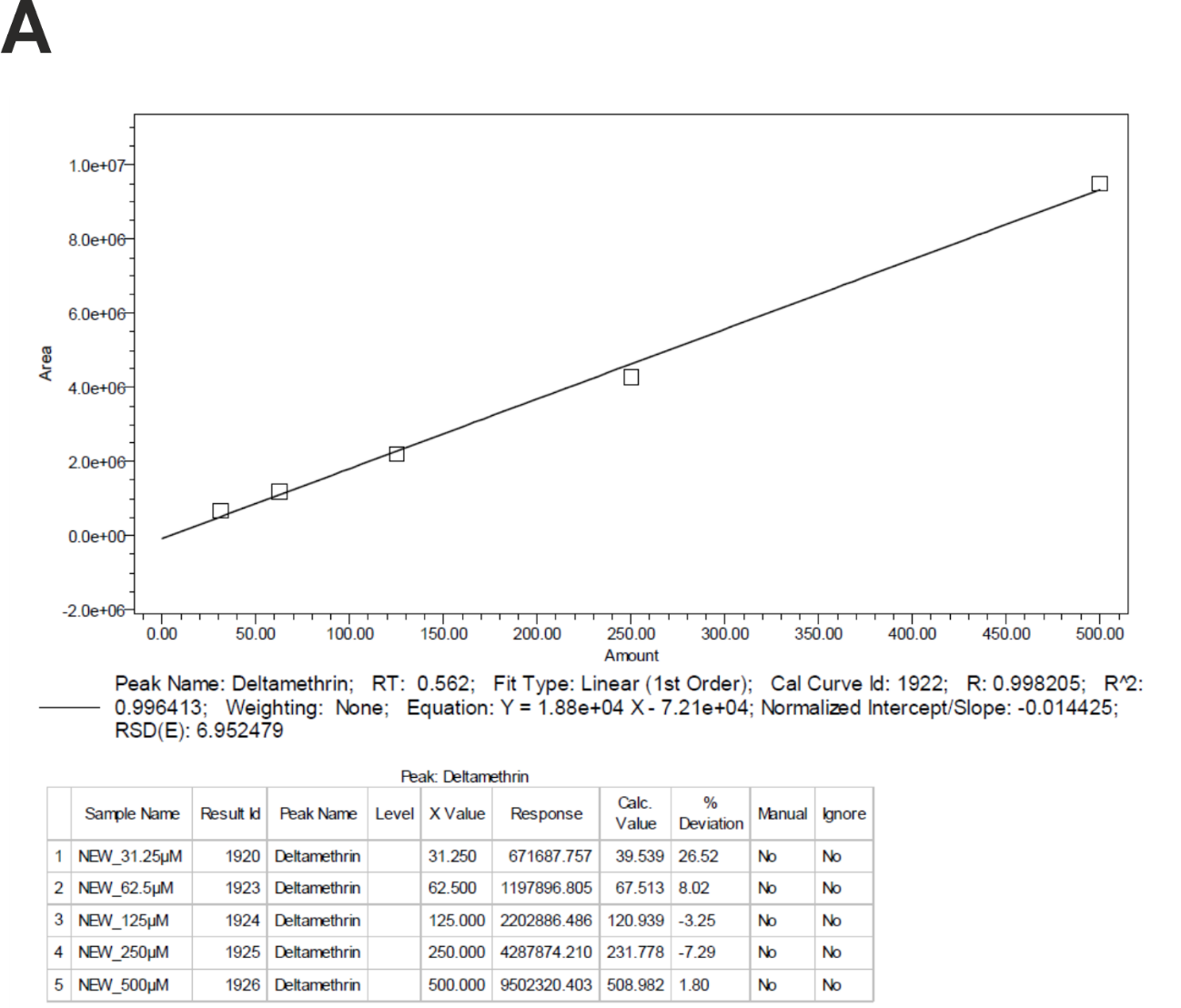


**Figure. 7: Results of Deltamethrin degradation by CSDIR protein.** (A) Standard curve of deltamethrin.


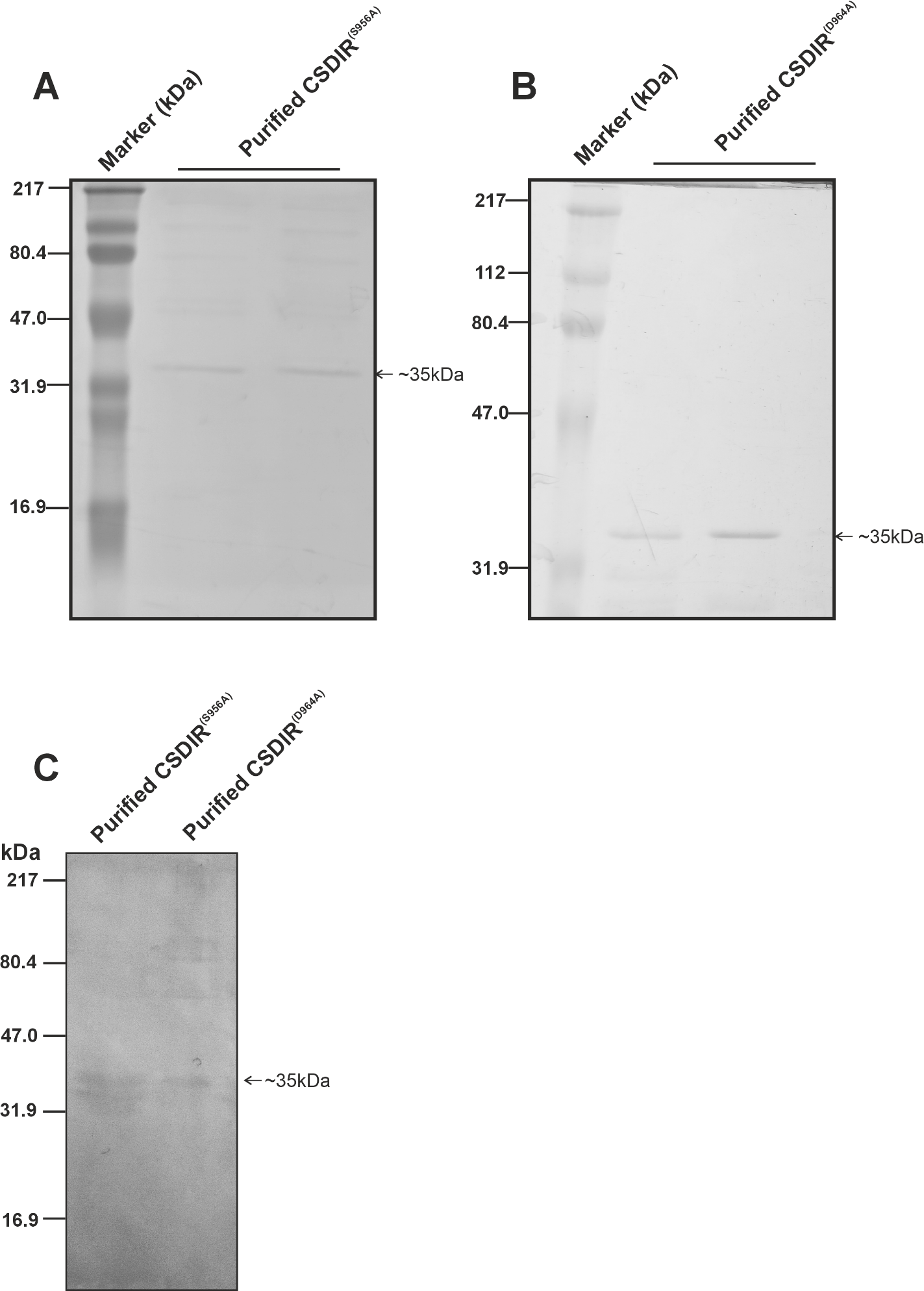


**Figure 8: Purification of CSDIR mutants.** (A) SDS-PAGE analysis of recombinant CSDIR^(S956A^) mutant in BL21-DE3 cells of *E. coli.* (B) SDS-PAGE analysis of recombinant CSDIR^(D964A)^ in BL21-DE3 cells of *E. coli.* (C) Western blot confirmation of purified CSDIR^(S956A^) and CSDIR^(D964A)^ mutant through Anti-his antibody.


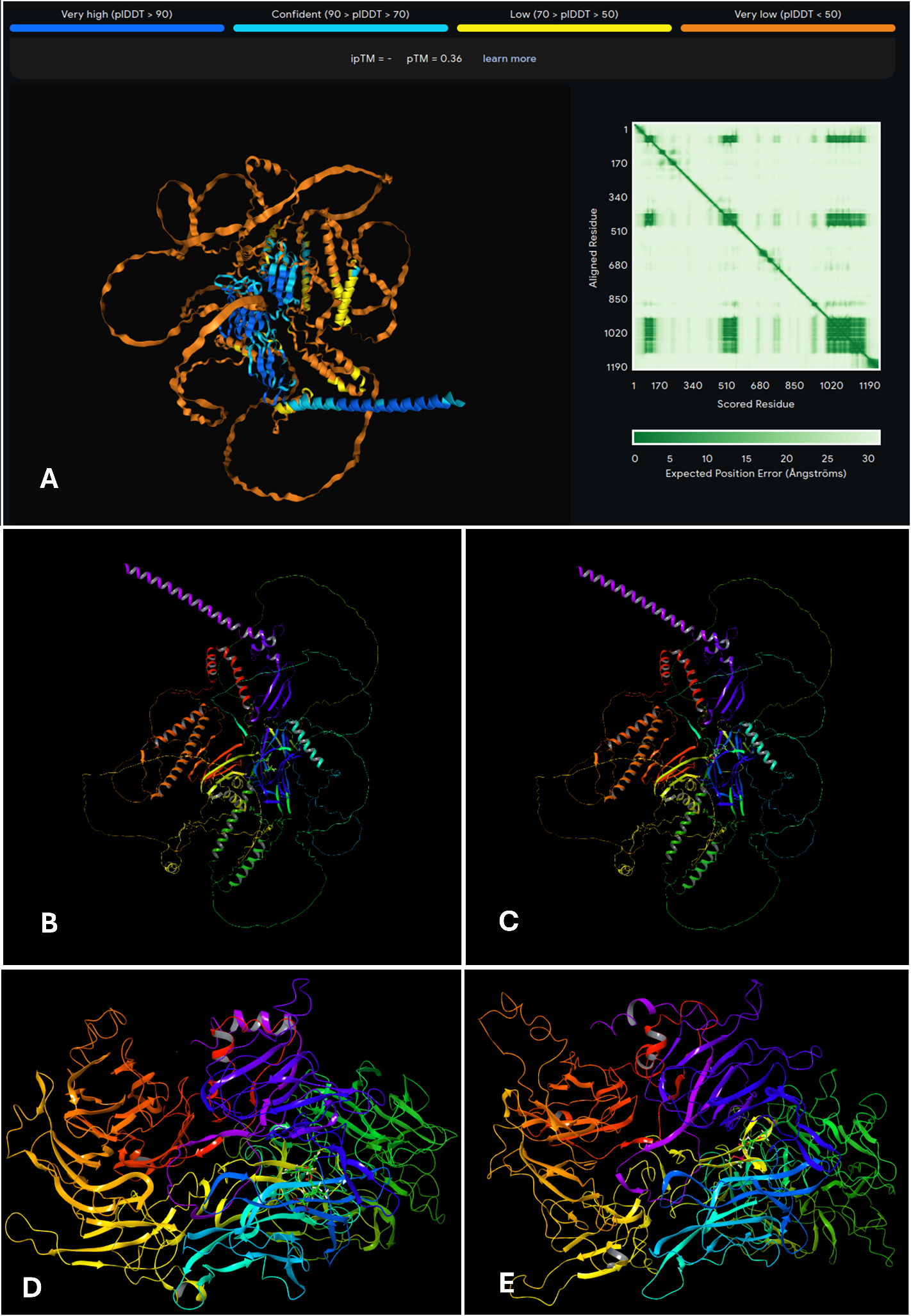


**Figure 9.** **Results of CSDIR modeling by AI (AlphaFold3).** (A) Alphafold3 model and scores for CSDIR model. (B) AlphaFold 3 model of CSDIR & (D) I-TASSER model of CSDIR docked with Malathion were superimposed. The ligand binding site is completely unmodelled in the Alphafold3 model which we have validated by SPR and Mutational studies. (C) AlphaFold 3 model of CSDIR & (E) I-TASSER model of CSDIR docked with Deltamethrin were superimposed. The ligand binding site is completely unmodelled in the Alphafold3 model which we have validated by SPR and Mutational studies.

# DETAILED LC-MS/MS PARAMETERS

**General reverse phase nano-Liquid Chromatography setting: Instrument name: Thermo Scientific Easy-nLC 1200 Amount of peptide loaded on column:** ~2 µg

**Analytical Column name:** PepMap RSLC C18 2µm, 75µm x 50cm (Thermo scientific)

**Column Temperature:** 60^0^ C

**A**: 98% water+2% Acetonitrile +0.1% Formic acid **B**: 20% water+80% Acetonitrile +0.1% Formic acid **Gradient**

| **TIME** | **DURATION** | **FLOW (nl/min)** | **%B** |
| --- | --- | --- | --- |
| 00:00 | 00:00 | 300 | 5 |
| 02:00 | 02:00 | 300 | 10 |
| 104:00 | 102:00 | 300 | 45 |
| 105:00 | 01:00 | 300 | 90 |
| 115:00 | 10:00 | 300 | 90 |
| 116:00 | 01:00 | 300 | 5 |
| 120:00 | 04:00 | 300 | 5 |

All solvents are of LCMS grade (Fischer Scientific).

Lock mass of 445.12003 Da was used for internal calibration.

**General Mass Spec setting:**

**Instrument name: Thermo Scientific Q Exactive Orbitrap**

***General setting:***

**Run Time**: 0 to 120 min **Polarity**: Positive **Default charge state**: 2 ***MS (MS1)setting:***

**Microscan**: 1

**Resolution**: 70000

**AGC (Automatic gain control)** 3e^6^ **Maximum IT (ion transfer) time**: 50 ms **No. of scan ranges**: 1

**Scan ranges:** 350 to 2000m/z

**Spectrum obtained was in profile mode**.

***MS MS (MS2) setting:***

**Microscan**: 1

**Resolution**: 17500

**AGC (Automatic gain control)** 1e^5^

**Maximum IT (ion transfer)** 120 ms

**Loop count**: 10 (top 10 masses will be fragmented one by one) **Maximum number of precursor to be plexed in single event**: 1 **Isolation window**: 1.5 m/z

**Isolation offset**: 0.0 m/z **Scan range:** 200 - 2000m/z **Fixed first mass**: 100 m/z

**Normalized collision energy**: 27 ***Data dependent settings* Minimum AGC target**: 1.00e^2^ **Charge exclusion**: unassigned **Dynamic exclusion time**: 50.0 S

**Proteome Discoverer 2.4**

Database – Proteins available for the organism Anopheles Stephensi on UniProt

**Link**: https://[www.uniprot.org/uniprotkb?query=(taxonomy_id:30069)](http://www.uniprot.org/uniprotkb?query=(taxonomy_id%3A30069))

**Methods and Parameters set:**

Max. allowed missed cleavage=2 Min. peptide length for search = 6 Maximum peptide length = 144 Precursor Mass tolerance = 10 ppm Fragment mass tolerance = 0.02 Da

Static Modification= Carbamidomethyl / +57.021 Da (C)

Target FDR (false discovery rate) = 0.01 (target decoy PSM validator)
